# Triplet‐triplet Annihilation Dynamics of Naphthalene

**DOI:** 10.1002/chem.202200781

**Published:** 2022-06-21

**Authors:** Mahesh Gudem, Markus Kowalewski

**Affiliations:** ^1^ Department of Physics Stockholm University Albanova University Centre SE-106 91 Stockholm Sweden

**Keywords:** *ab initio* calculations, conical intersections, polaritonic chemistry, quantum chemistry, quantum dynamics

## Abstract

Triplet‐triplet annihilation (TTA) is a spin‐allowed conversion of two triplet states into one singlet excited state, which provides an efficient route to generate a photon of higher frequency than the incident light. Multiple energy transfer steps between absorbing (sensitizer) and emitting (annihilator) molecular species are involved in the TTA based photon upconversion process. TTA compounds have recently been studied for solar energy applications, even though the maximum upconversion efficiency of 50 % is yet to be achieved. With the aid of quantum calculations and based on a few key requirements, several design principles have been established to develop the well‐functioning annihilators. However, a complete molecular level understanding of triplet fusion dynamics is still missing. In this work, we have employed multi‐reference electronic structure methods along with quantum dynamics to obtain a detailed and fundamental understanding of TTA mechanism in naphthalene. Our results suggest that the TTA process in naphthalene is mediated by conical intersections. In addition, we have explored the triplet fusion dynamics under the influence of strong light‐matter coupling and found an increase of the TTA based upconversion efficiency.

## Graphical abstract

**Triplet‐triplet annihilation** (TTA) of naphthalene has been investigated by using multi‐reference methods along with quantum dynamics simulations. The study involves obtaining the mechanistic details of the process and understand the time evolution of nuclear wavepacket on the computed potential energy surfaces. The results suggest that the TTA is mediated by conical intersections (CIs). It is also demonstrated that the strong light‐matter interaction increases the efficiency of TTA based photon upconversion.

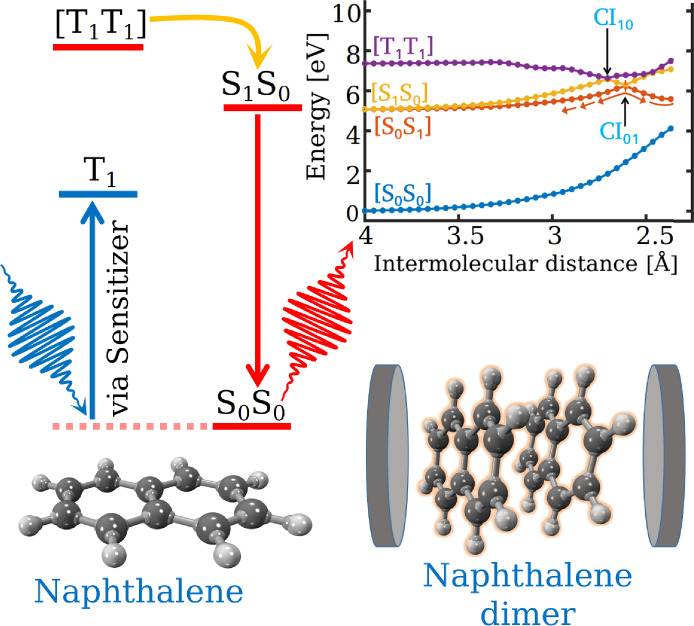

## Introduction

Triplet‐triplet annihilation (TTA), also termed as triplet fusion, is a bimolecular process in which two low‐energy triplet excited states convert into one high‐energy singlet, triplet or quintet state.^[1]^ As shown by several experimental studies on TTA, the formation of high energy singlet excited state is more likely than the other states with different spin multiplicity.[^2^, ^3^, ^4^, ^5^, ^6^, ^7^] The fusion of two low‐energy triplet sates to produce a single high‐energy photon in TTA process results in the delayed fluorescence, which was reported for the first time in anthracene derivatives around half a century ago.[^8^, ^9^, ^10^, ^11^] The two molecular species involved in this upconversion process are referred to as sensitizer and annihilator. Sensitizer species absorb photons in the visible‐to‐near‐IR region and have high intersystem crossing (ISC) yields to form the long‐lived triplet states. These properties of the sensitizer are highly required to efficiently populate the T_1_ state of annihilator molecule.

**Figure 1:**
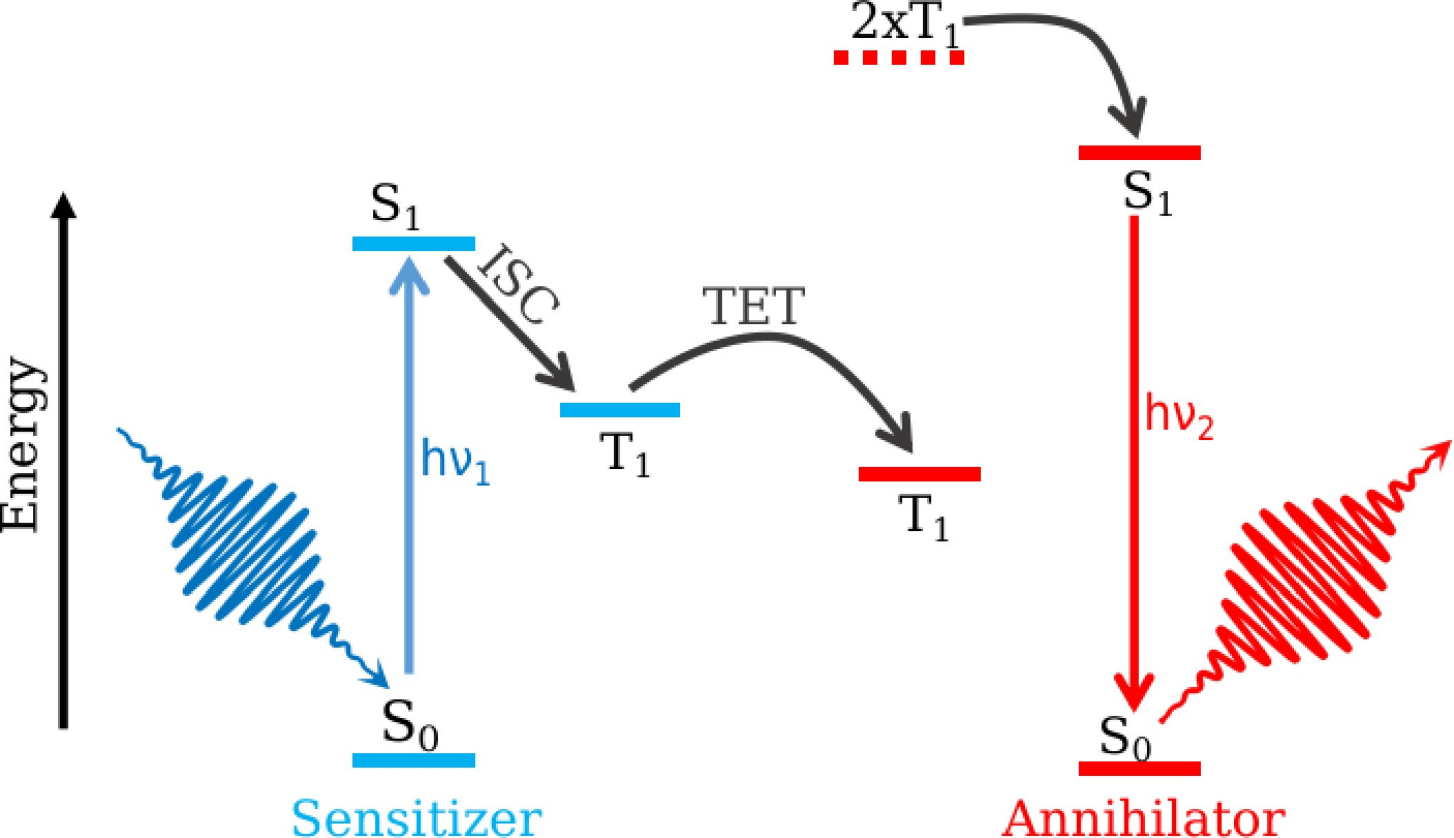
A schematic showing the Triplet‐triplet annihilation (TTA) based photon upconversion. S_1_ excitation of sensitizer with the incident light followed by the intersystem crossing (ISC) to the T_1_ state. Dexter triplet‐triplet energy transfer (TET) results in the population transfer to the T_1_ state of annihilator. Two annihilator molecules populating the T_1_ state couple (2xT_1_) and form the S_1_ state, which emits the high energy photon by radiatively decaying to the ground state.

The photon upconversion process via TTA is schematically shown in Figure 1, where multiple energy transfer steps are involved. Irradiating the sensitizer with low‐energy photons (h*ν*_1_) causes the electronic excitation to the S_1_ state, followed by the ISC to the T_1_ state. The population then transfers to the T_1_ state of the annihilator molecule via Dexter triplet‐triplet energy transfer (TET). The T_1_ states of two annihilator molecules fuse together to form the S_1_ state, which produces the high energy photon, h*ν*_2_ as a result of the fluorescence emission.^[12]^ Even though the TTA based photon upconversion has been employed in several areas like bio‐imaging,[^13^, ^14^, ^15^, ^16^, ^17^] photovoltaics,[^18^, ^19^, ^20^, ^21^, ^22^, ^23^, ^24^, ^25^, ^26^] photo‐dynamic therapy[^27^, ^28^] and solar fuels,[^3^, ^29^, ^30^, ^31^] the efficiency close to the maximum 50 % quantum yield is yet to be realized. Based on a few basic requirements corresponding to the involved molecular species, various design strategies have been proposed to minimize the loss channels and increase the TTA upconversion efficiency.[^12^, ^26^] The same design principles however, have shown to be producing very small quantum yields, less than 5 % in some cases.[^17^, ^32^, ^33^, ^34^] In order to further improve the design parameters and predict the better annihilators, few studies have used *ab initio* and TD‐DFT computed singlet‐triplet energy gaps.[^35^, ^36^, ^37^] Nevertheless, a complete molecular level understanding of the TTA mechanism, which provides the fundamental insights on the upconversion process, is still lacking.

The annihilator molecules developed so far are mostly based on a few parent molecular structures.[^26^, ^31^] Here we study naphthalene as one of the simplest parent molecules. Its derivative, TIPS(1,4‐bis((triisopropylsilyl)ethynyl))‐naphthalene has been reported to be undergoing the TTA induced upconversion with an efficiency of 20.5 %.^[38]^ It is noteworthy that the TIPS‐naphthalene is a recently (in 2020) developed annihilator molecule, whereas the structurally similar anthracene derivatives are the oldest documented TTA compounds.[^8^, ^9^, ^10^, ^11^] Furthermore, this naphthalene derivative along with Ir(C6)_2_(acac) sensitizer exhibits the record efficiency in visible‐to‐UV conversion.^[38]^ In recent studies, anthracene derivatives have also been found to be very efficient TTA compounds in near‐Infrared to blue photon upconversion.[^39^, ^40^] In the present work, we have intended to obtain the fundamental understanding of TTA based upconversion process by theoretically exploring the triplet fusion dynamics of naphthalene.

Few theoretical studies on the excited states of the naphthalene dimer exist which are limited to the singlet and triplet excitation energies of different conformers, and the understanding of the effects of intermolecular distance and orientation on the exciton splitting.[^41^, ^42^, ^43^, ^44^, ^45^, ^46^] Abrahamsson and co‐workers have shown the importance of singlet and triplet potential energy curve (PEC) shapes on the upconversion by using TD‐DFT calculations.^[47]^ In this study, we investigate the TTA process by considering naphthalene as a model system. Multi‐reference electronic structure methods along with nuclear wave packet dynamics have been employed to discern the fate of the annihilator after reaching the fused triplet‐triplet (2xT_1_ in Figure 1) state. Our goal is to explore the mechanistic details of TTA and the subsequent excited state pathways for a naphthalene dimer. The branching ratio between different excited state channels has been obtained from the dynamics simulations. In addition we have also studied the cavity influences on the photon upconversion efficiency of naphthalene.

Strong light‐matter coupling of electronic states has been shown to influence excited state chemical processes by manipulating the potential energy surfaces (PESs). Molecular electronic states in the presence of an optical cavity can couple to the cavity vacuum field and form hybrid light‐matter states called polaritons. Plenty of chemical reactions including photodissociation, photoisomerization, and electron transfer processes have been found to be altered by the presence of polaritonic states.[^48^, ^49^, ^50^, ^51^, ^52^, ^53^, ^54^, ^55^, ^56^, ^57^] In a theoretical study by Gu and Mukamel, coupling of a single molecule with the cavity mode has been shown to be suppressing the singlet fission, spin‐allowed conversion of singlet into two triplet states; reverse process of TTA, in pentacene dimers.^[58]^ More recently, Börjesson and co‐workers have demonstrated the conversion of an endothermic TTA process into an exothermic one by using the strong cavity‐molecule coupling.^[59]^ Based on these results, we have simulated the polaritonic‐nonadiabtic dynamics of naphthalene dimer by incorporating the light‐matter interaction in the corresponding Hamiltonian.

In this work, we confined ourselves to study the TTA process of naphthalene involving singlet electronic states; i. e., the role of high spin (triplet and quintet) states affecting the triplet fusion dynamics has been neglected. In addition, the influence of other loss channels, such as dimerization between excimer and ground state naphthalene, on the efficiency of TTA based upconversion should eventually be explored in the future studies.

The outline for the rest of the paper is as follows. Section II (Theory and Methods) presents the theoretical framework which includes the electronic structure methods, model Hamiltonian, and wave packet dynamics details in three subsections, respectively. Section III (Results and Discussion) is divided into four subsections. The first subsection deals with the excitation energies and optimized critical points. Different excited state pathways are discussed in the second subsection. The dynamics simulation results are presented in third (bare molecule) and fourth (coupled cavity‐molecular system) subsections. Conclusions are provided in section IV.

## Theory and Methods

### Electronic structure methods

The optimized geometries, energy pathways connecting the critical points (stationary and surface crossing points), and two‐dimensional potential energy surfaces have been obtained by using the complete active space self‐consistent field (CASSCF)[^60^, ^61^, ^62^] method along with the 6‐31G** basis set. No symmetry constraints were imposed on electronic structure calculations.

The crucial part in the successful application of the CASSCF method involves the selection of suitable active orbitals that describe the whole reaction process adequately. The ideal active space in the case of the naphthalene dimer would be 20 electrons in 20 orbitals [CAS(20/20)], which includes all *π*‐orbitals. However, employing such a large active space to explore the TTA process is computationally intractable and a reduced set of active orbitals has been considered instead. Based on the theoretical calculations of the four‐electron/four‐orbital model system H_4_,^[63]^ and the butadiene cyclo addition reaction,^[64]^ an excimer minimum (referred to as pericyclic minimum) was proposed during the TTA process of anthracene.^[65]^ The excimer minimum could decay either to the dimerized product or to the individual monomers in the ground state. Similar energy profiles can be anticipated for the TTA process of naphthalene. Our CASSCF calculations suggest that at least 12 *π*‐orbitals (six from each monomer) are essential to describe the complete TTA path of naphthalene involving the excimer minimum and the ground state dimerized product. One ring of each naphthalene monomer perturbs significantly during the formation of the dimerized product, whereas the other ring is unaffected. Accordingly, we have considered the active orbitals that are localized on a single ring of the monomer, though this reduced active space overestimates the vertical excitation energies. The final active space that we have used for all multi‐reference calculations of the naphthalene dimer is CAS(12/12) and the corresponding orbitals are shown in Figure S1 in Supporting Information. The active space for the monomer calculations consists of 6 electrons in 6 *π*‐orbitals that are localized on a single ring of naphthalene.

We have included the four lowest electronic states in the state‐averaged CASSCF (SA4‐CASSCF) wave function to obtain the vertical energies, the minimum energy path for TTA and the subsequent dimerization, and the two‐dimensional PESs of naphthalene dimer. The vertical excitation energies have also been computed at the SS‐CASPT2[^66^, ^67^] level of theory with an IPEA shift of 0.25 a.u, and compared them to the CASSCF values to validate the latter method. Additional CASSCF and SS‐CASPT2 vertical excitation energy computations were performed at the larger basis set 6‐311++G**. The equilibrium geometries of the reactant, where the two monomers are well separated, have been optimized at the SA4‐CASSCF level of theory. The electronic properties, non‐adiabatic couplings (Figure S2–S7 in the Supporting Information), and transition dipole moments (Figure S8–S10 in the Supporting Information), required for the dynamics simulations have been computed at SA4‐CASSCF level of theory. To optimize the other critical points, SA2‐CASSCF was used due to convergence issues with SA4‐CASSCF. A non‐radiative decay pathway from the excimer minimum to the ground state via S_1_/S_0_ CI has been computed at the SA2‐CASSCF level of theory. For the monomer calculations, SA3‐CASSCF (S_0_, S_1_ and T_1_) reference wave function with an active space of CAS(6/6) has been used. The MOLPRO‐2020 quantum chemistry program package has been used for all electronic structure calculations.[^68^, ^69^]

The non‐adiabatic couplings between three excited states have been computed by using the analytical method implemented in MOLPRO and are represented by a 3*N* dimensional vector where *N* denotes the number of atoms. Each element of the vector is described by $\left\langle \psi_{i}|\frac{\partial }{\partial x_{k}}\psi_{j}\right\rangle$ , where *ψ_n_* represents the electronic wavefunction and *x_k_* is the one of the Cartesian coordinate (*x*, *y*, *z*) of the *k* ^*th*^ atom. In the current study, the 3N dimensional non‐adiabatic coupling vector has been transformed to reaction coordinates *q*_1_ and *q*_2_, by using the following transformation:^[70]^

(1) $$f_{ij}^{\left(q_{1}\right)}=\sum_{k=1}^{3N}\frac{\partial x_{k}}{\partial q_{1}}\left\langle \psi_{i}|\frac{\partial }{\partial x_{k}}\psi_{j}\right\rangle$$

(2) $$f_{ij}^{\left(q_{2}\right)}=\sum_{k=1}^{3N}\frac{\partial x_{k}}{\partial q_{2}}\left\langle \psi_{i}|\frac{\partial }{\partial x_{k}}\psi_{j}\right\rangle$$

The partial derivatives of the Cartesian coordinates with respect to the reactive coordinates, $\frac{\partial x_{k}}{\partial q_{1}}$ and $\frac{\partial x_{k}}{\partial q_{2}}$ are computed using central finite differences, and the non‐adiabatic coupling elements $\left\langle \psi_{i}|\frac{\partial }{\partial x_{k}}\psi_{j}\right\rangle$ are taken from the MOLPRO calculation.

### Model Hamiltonian

The molecular Hamiltonian (${\hat{H}}_{M}$ ) for the time evolution of the nuclear wave function has been expressed in the adiabatic basis as,

(3) $$\begin{array}{ccc} {\hat{H}}_{M}=\; & -\frac{\hbar^{2}}{2}\sum_{i=1}^{2}\frac{1}{m_{i}}\frac{\partial^{2}}{\partial q_{i}^{2}}+\sum_{i=1}^{N_{e}}{\hat{V}}_{i}\left(q\right)\sigma_{ii}\sigma_{ii}^{†}\; & \;\\ \; & +\sum_{i=1}^{N_{e}}\sum_{j=1}^{N_{e}}{\hat{S}}_{ij}\left(q\right)\left(\sigma_{ij}+\sigma_{ij}^{†}\right)\;,\left(1-\delta ij\right);\; & \;\\ \; & \mathrm{where}\;\hat{S}ji=-\hat{S}ij\; & \; \end{array}$$

Here, the first term corresponds to the kinetic energy operator in which *m_i_* are the reduced masses of the corresponding reactive coordinates *q_i_. N_e_* denotes the number of electronic states considered in the dynamics simulations, and ${\hat{V}}_{i}$ represents the PES of the *i* ^*th*^ state, which depends on the nuclear degree of freedom **q**=(*q*_1_,*q*_2_)^*T*^. *σ_ij_* =$\left|\left.i\right\rangle \left\langle j\right.\right|$ and $\sigma_{ij}^{†}$ =$\left|\left.j\right\rangle \left\langle i\right.\right|$ are the annihilation and creation operators corresponding to the electronic excitation, respectively. ${\hat{S}}_{ij}$ (**q**) is the non‐adiabatic coupling between *i* and *j* electronic states, which has the following form:

(4) $$\begin{array}{c} \;{\hat{S}}_{ij}\left(q\right)=\sum_{k}\frac{1}{m_{k}}\left(f_{ij}^{\left(q_{k}\right)}\frac{\partial }{\partial q_{k}}+\frac{1}{2}\frac{\partial }{\partial q_{k}}f_{ij}^{\left(q_{k}\right)}\right) \end{array}$$

In this work, three electronic states have been considered to be active in the dynamics simulations, i. e., *N_e_* =3.

In addition to the dynamics of the bare molecule, we also have investigated the cavity effects on the triplet fusion dynamics of naphthalene by incorporating the light‐matter interaction terms in the Hamiltonian. The corresponding coupled cavity‐molecule Hamiltonian reads:

(5) $${\hat{H}}_{CM}={\hat{H}}_{M}+{\hat{H}}_{C}+{\hat{H}}_{I}\;$$

where ${\hat{H}}_{C}$ and ${\hat{H}}_{I}$ are the cavity and light‐matter interaction Hamiltonians, respectively. The quantized cavity mode Hamiltonian with frequency *ω_c_* is given by

(6) $$\begin{array}{c} \;{\hat{H}}_{C}=\hbar \omega_{c}\left({\hat{a}}^{†}\hat{a}+\frac{1}{2}\right)\;, \end{array}$$

where *a* and ${\hat{a}}^{†}$ annihilate and create an excitation in the photonic subspace, respectively. The cavity‐molecule interaction within the rotating wave approximation is described by

(7) $${\hat{H}}_{I}=\sum_{i=1}^{N_{e}-1}\sum_{j=i+1}^{N_{e}}\epsilon_{c}\mu_{ij}\left(q\right)\left(a^{†}\sigma_{ij}+a\sigma_{ij}^{†}\right)$$

where *μ_ij_* are the transition dipole moments between different electronic states. The strength of the electric field, *ε_c_* depends on the cavity mode volume *V*:

(8) $$\epsilon_{c}=\sqrt{\frac{\hbar \omega_{c}}{2\epsilon_{0}V}}$$

Note that the current model does not consider the cavity dissipation effects, i. e., lossless mirrors have been assumed.

### Quantum dynamics

The TTA dynamics of bare naphthalene, and the cavity coupled system have been simulated by numerically solving the time‐dependent Schrödinger equation with the Hamiltonians given in Eq. 3 and Eq. 5, respectively. The reactive coordinates *q*_1_ and *q*_2_ have been used to obtain the molecular kinetic energy term in Eq. 3. The nuclear wavepacket was propagated on the two‐dimensional potential energy grid by using the Chebychev method^[71]^ as implemented in our in‐house quantum dynamics code QDng. The time evolution of the wave packet was initiated by vertically placing the lowest vibrational state of the electronic ground state on the excited state PES of the naphthalene dimer. The initial nuclear wave function has been obtained by using the imaginary time propagation method.^[72]^ In order to avoid the reflections of the nuclear wave packets at the edge of the potential energy grid, a perfectly matched layer has been employed.^[73]^

The probability of the population reaching the upconversion region on a particular electronic state *i* is defined as the expectation value of the Heaviside step function $H\left(q_{1},q_{2}\right)$ with the time‐dependent nuclear wavefunction $\psi_{i}\left(t\right)$ .

(9) $$P_{i}^{UC}=\left\langle \psi_{i}\left(t\right)\left|H\left(q_{1},q_{2}\right)\right|\psi_{i}\left(t\right)\right\rangle$$

The Franck‐Condon (FC) area of the PESs has been considered to be the upconversion region. The corresponding step function is given by
(10)

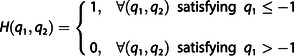




## Results and Discussion

### Vertical energies and optimized critical points

**Figure 2:**
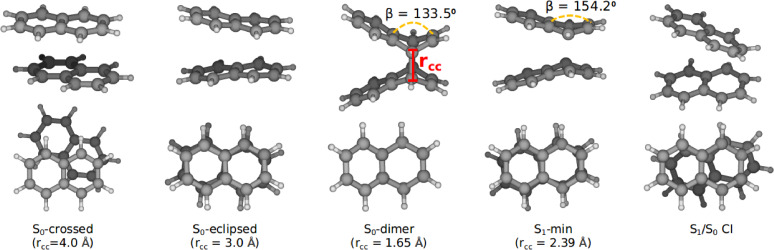
Critical points optimized on the PESs of naphthalene dimer. Both top and side views are given for clarity. The value in parentheses is represented by the intermolecular distance between naphthalene monomers. β with dashed yellow curve represents the butterfly bending angle.

The reactant minimum energy structure (S_0_‐crossed), which has been used to compute the vertical excitation energies of the naphthalene dimer and other optimized critical points are shown in Figure 2. We also have calculated the vertical energies of naphthalene monomer and correlated them with those of the dimer. The CASPT2 excitation energy for the S_1_ state of naphthalene has been found to be 5.06 eV, which is an overestimation to the best calculated vertical energy of 4.03–4.25 eV.[^74^, ^75^] The discrepancy can be attributed to the localized nature of the active orbitals employed in CASSCF and CASPT2 calculations (Figure S1 in Supporting Information). Among the 10 *π*‐orbitals, 6 orbitals that are mostly localized on a single ring of monomer have been considered. This particular selection is necessary to investigate the complete mechanistic details of the triplet‐triplet annihilation (TTA) process without causing the electronic structure calculations to become computationally prohibitive (see “Theory and Methods“ section for more details). The lowest triplet state lies below the S_1_ state with an excitation energy of 3.93 eV. Based on the computed energetics, naphthalene fulfills the primary requirement to undergo the TTA process, which is (2xE${}_{T_{1}}$ =7.86 eV)>(E${}_{S_{1}}$ =5.06). In the present work, we have constructed a two‐dimensional model to understand the triplet fusion dynamics of naphthalene. Since CASPT2 would create computational bottlenecks, CASSCF has been employed instead. The vertical energies computed at the CASSCF level of theory are showing a striking resemblance with those of the CASPT2 and MRCI (Table S1 in Supporting Information). Therefore, the CASSCF generated PESs describe the essential features of the TTA process in naphthalene. The excitation energies at the improved basis set 6‐311++G** are compiled in Table S2 in Supporting Information. A close agreement of these values with the 6‐31G** ones suggest that the smaller basis set is sufficient to describe the investigated excited states of naphthalene.

**Figure 3:**
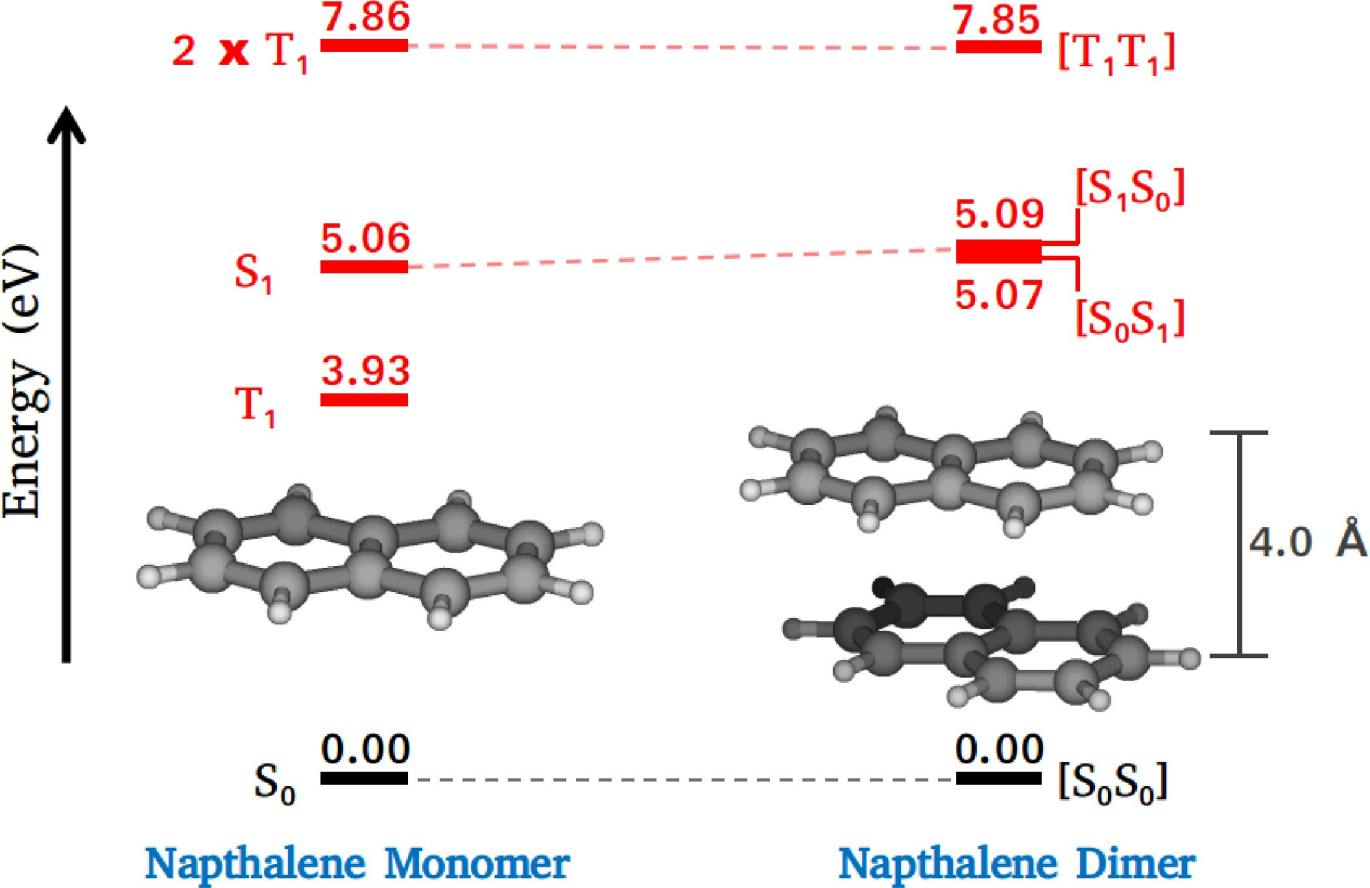
State correlation between monomer and dimer of naphthalene at the S_0_‐crossed geometry obtained at the CASPT2/6‐31G** level of theory.

**Figure 4:**
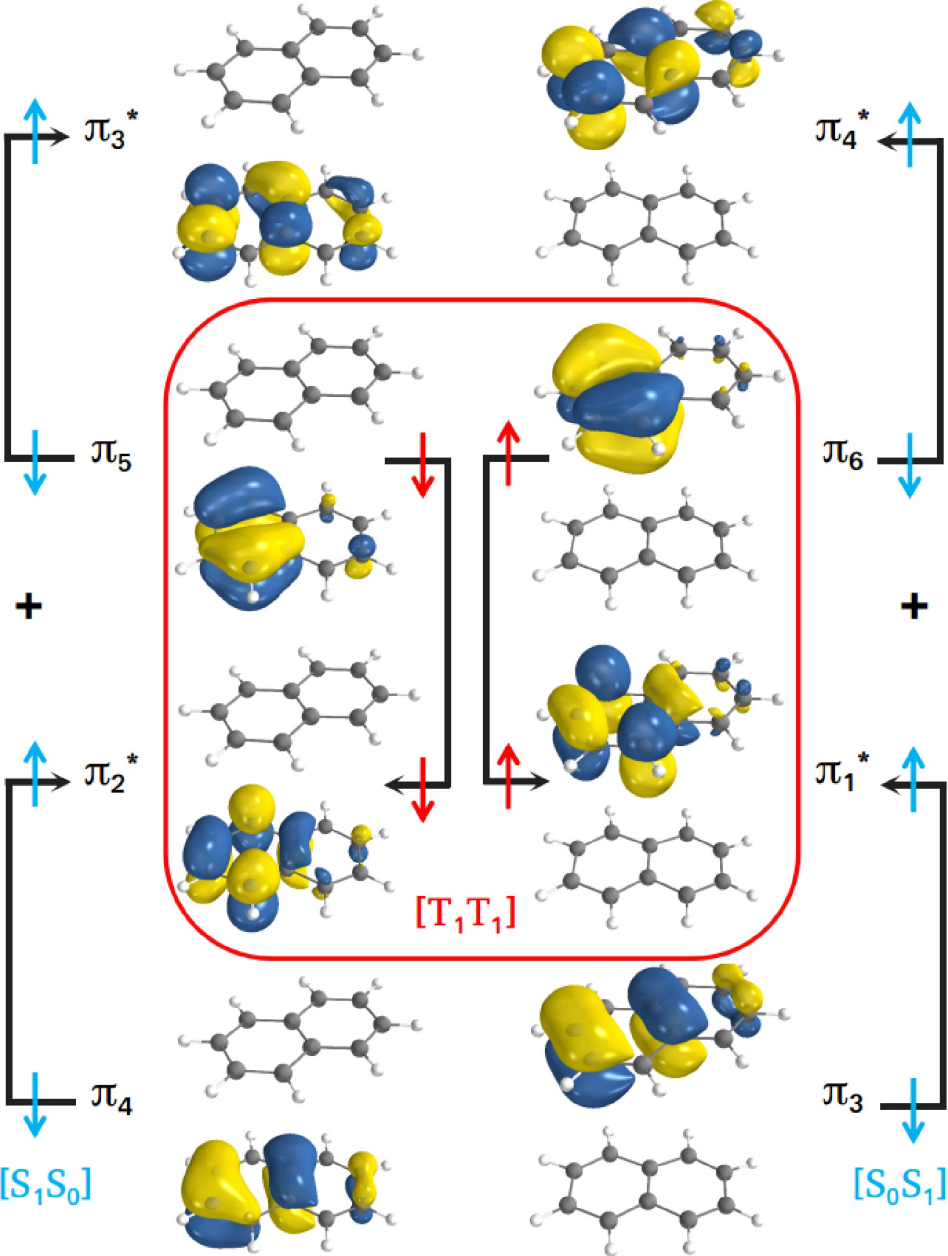
Frontier molecular orbitals involved in the electronic transition for [S_1_S_0_], [S_0_S_1_] and [T_1_T_1_] states. Red (Cyan) colored arrows are used to represent the orbital occupations and the electronic spins of [T_1_T_1_] ([S_1_S_0_] and [S_0_S_1_]) state (states).

The estimated vertical energies at the FC geometry suggest that the triplet‐triplet state of the dimer corresponds to the third singlet excited state, which is labelled as [T_1_T_1_] in the state‐correlation diagram (Figure 3). Both the naphthalene monomers are in T_1_ and the state overall has singlet character. A similar notation has been used to represent the other electronic states of the dimer; [S_1_S_0_], for instance, is for the state where one monomer is in the ground state and the other in the first excited state. The S_1_ state in [S_1_S_0_] ([S_0_S_1_]) has been characterized by a linear combination of $\pi_{5}\to \pi_{3}{}^{*}$ and $\pi_{4}\to \pi_{2}{}^{*}$ ($\pi_{6}\to \pi_{4}{}^{*}$ and $\pi_{3}\to \pi_{1}{}^{*}$ ) orbital transitions, which is in agreement with the previous theoretical studies on the photo‐physics of naphthalene monomer.^[74]^ The orbitals characterizing the [T_1_T_1_] state are *π*_5_, $\pi_{2}{}^{*}$ , *π*_6_ and $\pi_{1}{}^{*}$ , among which the first two orbitals are localized on one monomer and the last two are on the other monomer. The triplet‐triplet state wavefunction has also been found to be a superposition of multiple determinants. One of the leading configuration is shown in Figure 4, where two electrons in *π*_5_ and $\pi_{2}{}^{*}$ have down spin, and the electrons of *π*_6_ and $\pi_{1}{}^{*}$ have up spin. The two lowest singlet excited states ([S_1_S_0_] and [S_0_S_1_]) of naphthalene dimer are degenerate at the S_0_‐crossed geometry (Figure 3), which can be explained by their identical electronic nature as depicted in Figure 4. Similar to the case of monomer, the excitation energies of the dimer at CASSCF level of theory match the CASPT2 values at both 6‐31G** and 6‐311++G** basis sets (Tables S1 and S2 in Supporting Information). This reaffirms that CASSCF/6‐31G** is appropriate for investigating the triplet fusion process of naphthalene.

We have optimized the relevant critical points on the PESs of the naphthalene dimer. The equilibrium geometry corresponding to the reactant complex of the dimer has been obtained by performing the constrained optimization with a constraint intermolecular distance r_*CC*_ of 4.0 Å. The naphthalene monomers in the optimized structure are not stacked perfectly, instead they show a parallel displacement and rotation. The corresponding geometry is denoted as S_0_‐crossed in Figure 2. Starting from a geometry with small r_*CC*_ (around 2 Å), we could optimize the dimerized product (S_0_‐dimer) on the ground state without imposing any geometrical constraints. Three primary differences have been observed between the S_0_‐dimer and S_0_‐crossed structures: 1) the dimer is structurally more symmetric and the monomers are perfectly stacked. 2) The intermolecular distance, r_*CC*_ reduces to 1.65 Å due to the formation of two sigma bonds between the monomers. 3) The newly formed sigma bonds change the hybridization of the corresponding carbon atoms from sp^2^ to sp^3^. Consequently, the β (butterfly bending) angle reduces from 180° to 133.5°, which results in a bent at the sp^3^ carbon atoms (see Figure 2). The geometrical differences suggest that the dimerization process starting from the reactant complex first involves the formation of a perfectly stacked conformer due to the *π*‐*π* stacking interaction between the monomers. This can also be confirmed by obtaining another constrained minimum (S_0_‐eclipsed) geometry with the reduced constraint value of r_*CC*_=3 Å. A minimum energy structure has been optimized for the first excited state (S_1_‐min), which exhibits similar geometrical changes as S_0_‐dimer but to a lesser extent resulting the reduction in r_*CC*_ to 2.39 Å and β to 154.2°. In addition to the discussed stationary points, we also have optimized a conical intersection between ground and the first excited state, denoted as S_1_/S_0_ CI. Among the obtained critical points, the S_1_/S_0_ CI has been found to be the most asymmetric structure with respect to S_1_‐min (or S_0_‐dimer). This CI allows the molecule on the first excited state to decay to the ground state non‐radiatively. Similar type of CI was observed in the case of butadiene dimerization process.^[76]^

### Excited‐state pathways from [T_1_T_1_]

**Figure 5:**
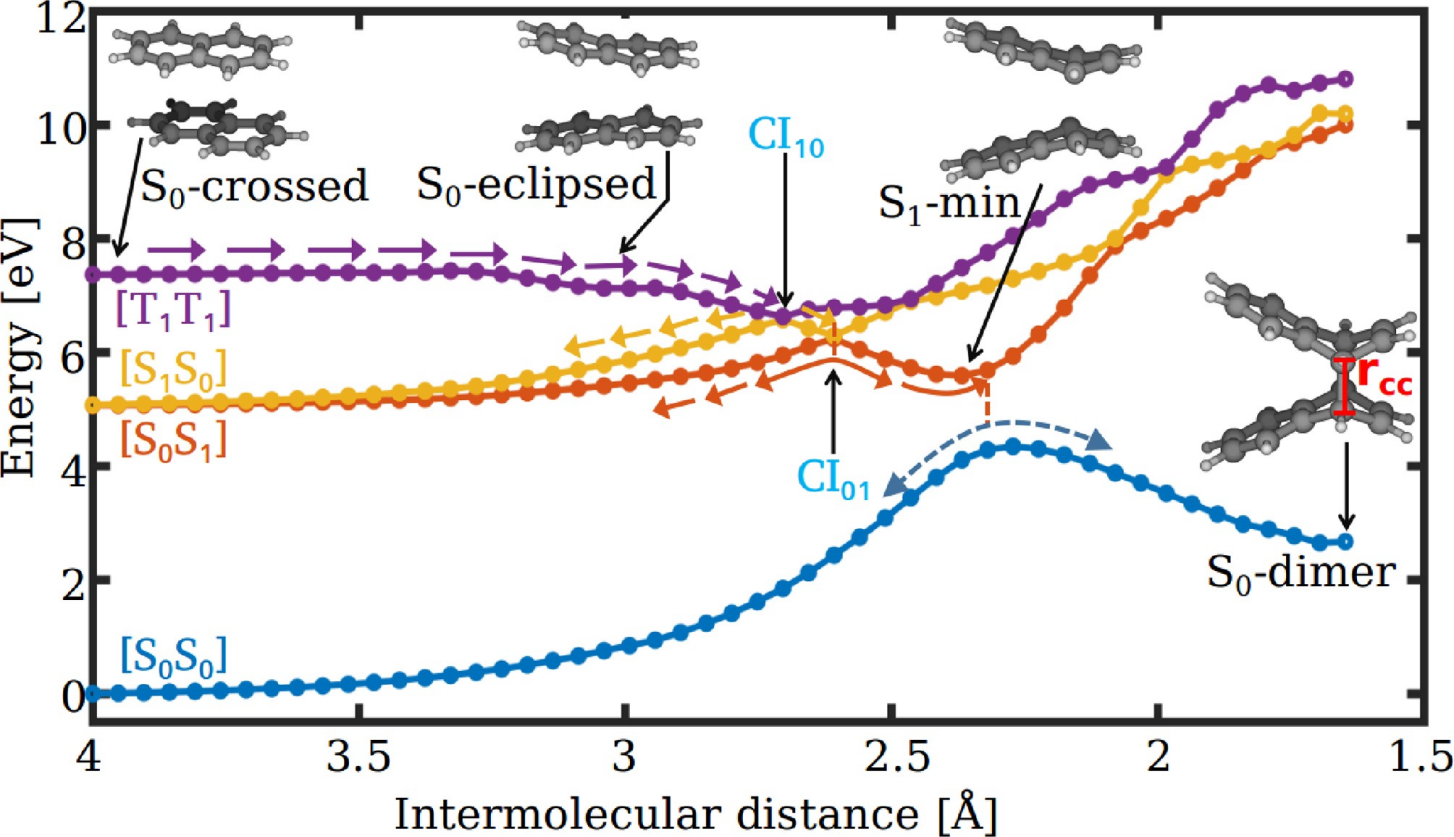
Triplet‐triplet annihilation followed by the dimerization pathway of naphthalene dimer computed at CASSCF/6‐31G** level of theory. CI_10_ and CI_01_ refer to the crossings between [T_1_T_1_] and [S_1_S_0_], and [T_1_T_1_] and [S_0_S_1_] states, respectively.

The fate of the molecule after reaching the [T_1_T_1_] state has been scrutinized by connecting the above‐discussed critical points of naphthalene dimer. Starting from the reactant complex (S_0_‐crossed), a relaxation pathway toward S_0_‐dimer has been constructed by performing three sets of geometry interpolations: one from S_0_‐crossed to S_0_‐eclipsed, two between S_0_‐eclipsed and S_1_‐min, and three from S_1_‐min to S_0_‐dimer. Subsequently, at these interpolated geometries, the CASSCF energies have been calculated for the four lowest electronic states. The corresponding potential energy curves are depicted in Figure 5. Interestingly, the obtained energy profile is remarkably similar to the one proposed in the case of anthracene dimerization.^[77]^ It is clear from the figure that the [T_1_T_1_] state of naphthalene intersects with both the [S_1_S_0_] and [S_0_S_1_] states along the dimerization pathway. These crossings will be referred to as CI_10_ and CI_01_, respectively, in the rest of the paper. The path also exhibits an avoided crossing between ground and first excited state in the vicinity of S_1_‐min. CI_10_ and CI_01_ are accessible for the S_0_‐crossed geometry on the [T_1_T_1_] state through a barrierless pathway (Figure 5). The T_1_T_1_ state population is consequently transferred into the lower electronic states efficiently. Furthermore, there are two branches possible at these degenerate points: one towards the S_1_‐min and the other towards the formation of reactant complex on [S_1_S_0_] or [S_0_S_1_] states. The population reaching the minimum on the first excited state could decay to the ground state via avoided crossing and forms either dimerized product or the ground state reactant complex. On the other hand, the population transfer towards the S_0_‐crossed geometry on the [S_1_S_0_] and [S_0_S_1_] states undergo spontaneous emission with a higher frequency than the incident light. By using the Einstein spontaneous emission coefficient, the fluorescence lifetime of naphthalene dimer at S_0_ eclipsed geometry has been estimated to be 550 ns, which is qualitatively similar to the experimentally reported value around 100 ns in cyclohexane solution.^[78]^ In this upconversion mechanism, two low energy triplet excitons of the [T_1_T_1_] state annihilate to form one high energy singlet exciton of [S_1_S_0_] (or [S_0_S_1_]) state, hence will be referred to as the TTA based upconversion process. A curve crossing between singlet and [T_1_T_1_] states was also revealed in a singlet fission and TTA study of ethylene dimer model by using *ab initio* and DFT calculations.^[79]^

We want to point out that the cascade of energy transfer steps (see Figure 1) involved in the triplet fusion are less favorable in the gas phase. The higher efficiency of upconversion is often observed in liquid/quasi‐solid state systems, mainly due to the large triplet exciton mobility of the sensitizer that favors the TET process. Some technical applications of TTA require rigid and solvent‐free conditions, thereby the solid‐state TTA compounds are of significant interest.[^80^, ^81^]

**Figure 6:**
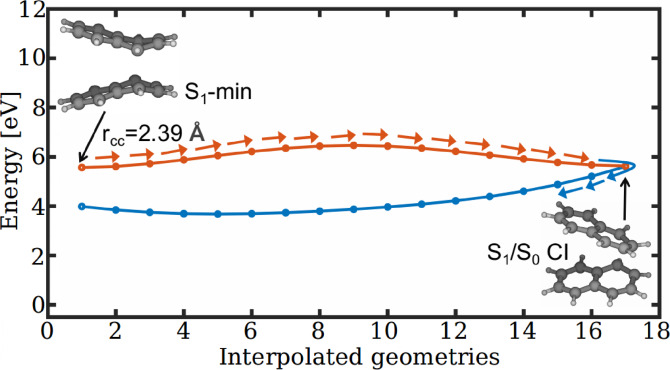
Non‐radiative decay pathway from S_1_‐min to the ground state via S_1_/S_0_ CI calculated at the CASSCF/6‐31G** level of theory.

The *π*‐*π* interactions between the stacked monomers in the [T_1_T_1_] state result the formation of S_1_‐min. From this pericyclic minimum geometry, the molecule has a possibility for another non‐radiative decay pathway through S_1_/S_0_ CI. Although the corresponding interpolated path (Figure 6) displays a small barrier, it is still energetically accessible due to the relatively high energy at the FC point.

### Dynamics of triplet‐triplet annihilation

As we discussed in the previous subsection, the molecule in the [T_1_T_1_] state not only undergoes the TTA process but also forms the pericyclic minimum in the first excited state which subsequently decays to the ground state. In order to understand the branching ratios between different decay channels for the [T_1_T_1_] state, quantum dynamics simulations have been performed. We first discuss the construction and topological features of two‐dimensional PESs describing the triplet fusion of naphthalene, and eventually present the simulation results.

**Figure 7:**
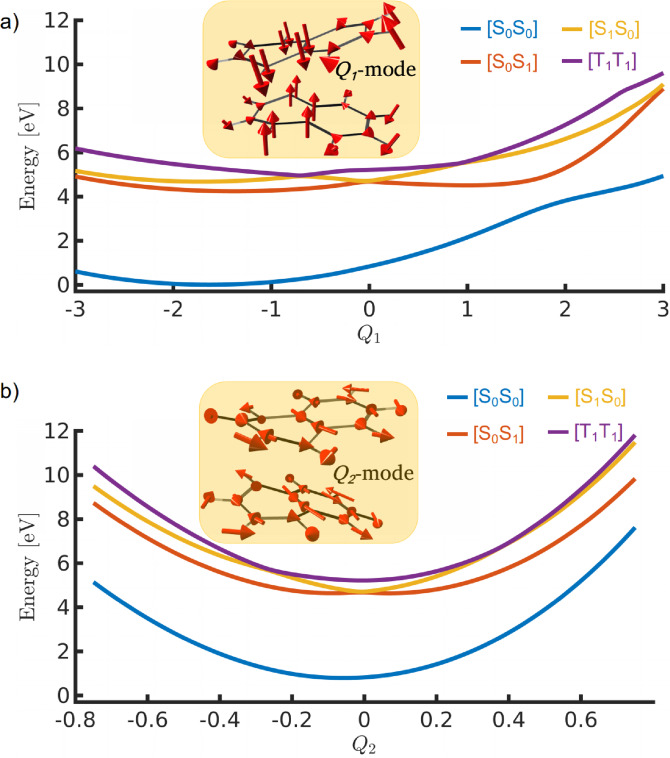
Potential energy curves along *Q*_1_ and *Q*_2_ modes at the CASSCF/6‐31G** level of theory. The corresponding vectors have also shown. CI_01_ has been used as the reference geometry.

It is evident from Figures 5 and 2 that the TTA process mainly involves two internal modes; the butterfly motion, which results in the bending of the newly formed C−C bonds, and an intermolecular motion that reduces the distance between naphthalene monomers. The combined coordinate of these two nuclear modes affects the energy difference between the different electronic states and forms CI_10_ and CI_01_. At any CI, one can distinguish two orthogonal vectors, called branching space vectors, along which the degeneracy between two electronic states will be lifted. Therefore, at least two‐dimensional PES models are necessary to describe the essential features of photochemical reactions involving CIs. To ascertain the two important coordinates corresponding to the TTA process of naphthalene, we have computed the gradient difference and derivative coupling vectors for the CI_01_ geometry. Based on the analysis of the calculated branching space vectors, three normal modes, intermolecular stretching, butterfly motion, and symmetric ring stretching have been used to represent the two reaction coordinates (denoted as *Q*_1_ and *Q*_2_) of TTA. A linear combination of the former two normal modes leads to the formation of *Q*_1_, and the last mode has been considered to be *Q*_2_ (see Figure 7). The calculated potential energy curves along *Q*_1_ and *Q*_2_ (Figure 7) indicate that the CI degeneracy indeed breaks along the considered reaction coordinates. As expected, the energy profile of *Q*_1_ (Figure 7(a)) is in good agreement with the energy path in Figure 5. The significant difference between the two profiles has been observed at large intermolecular distances. The energy curves are almost constant for large r_*CC*_ in Figure 5, whereas they increase in the corresponding region of the curves in Figure 7(a). This is due to the presence of the butterfly motion in the *Q*_1_ mode, which affects the potential energy even for large intermolecular distances. As a result, the PECs exhibit minima for negative *Q*_1_ values.

**Figure 8:**
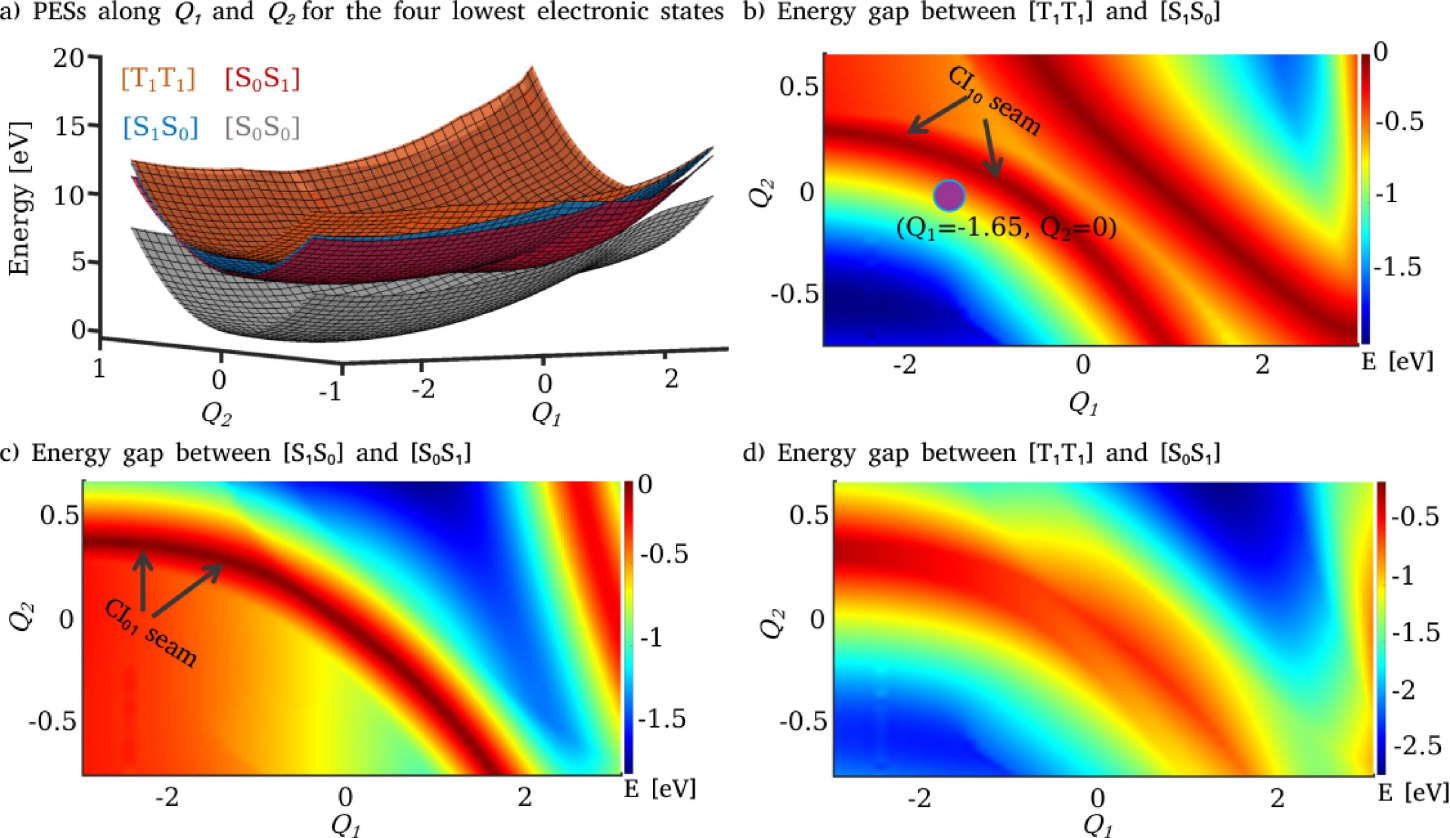
a) Two‐dimensional PESs for the four lowest electronic states of naphthalene dimer along *Q*_1_ and *Q*_2_ internal coordinates calculated at CASSCF/6‐31G** level of theory. Energy gap between different electronic states are represented in b), c) and d). Violet colored filled circle in (b) represents the position of the initial wave packet in the space of *Q*_1_ and *Q*_2_ coordinates.

Figure 8(a) displays the adiabatic PESs (constructed by using CI_01_ as the reference geometry) for the four lowest electronic states of naphthalene dimer in a two‐dimensional space spanned by *Q*_1_ and *Q*_2_ molecular modes. The energy gap between different excited state surfaces is shown to identify the region of intersection points (Figure 8(b), (c) and (d)). The surfaces exhibit seams of degenerate points between the adjacent electronic states (Figure 8(b) and (c)). Since the ground state surface lies sufficiently far away in energy from the other three excited states, it was not included in the quantum dynamic simulations. The couplings between the three excited states have been incorporated by calculating the intrastate non‐adiabatic couplings. The primary goal of the dynamics simulation study is to understand the branching ratio between different decay channels of the [T_1_T_1_] state via CI_10_ and CI_01_ crossings. Our model does not include the couplings of the excited states with the ground state, and the fate of the molecule after arriving at the pericyclic minimum cannot be determined from the current simulations. Moreover, higher dimensional PESs with additional vibrational modes, describing the non‐radiative decay pathway (Figure 6), are necessary to deal with the decay dynamics of S_1_‐min to the ground state. The timescale of the population dynamics cannot be directly compared with the experimental data, because a perfectly stacked dimer in the [T_1_T_1_] state is the starting point for the simulation, which is not the case for an experimental study. Another major distinction between the current study and an experiment is that the liquid/solid phase effects have not been considered in the model.

**Figure 9:**
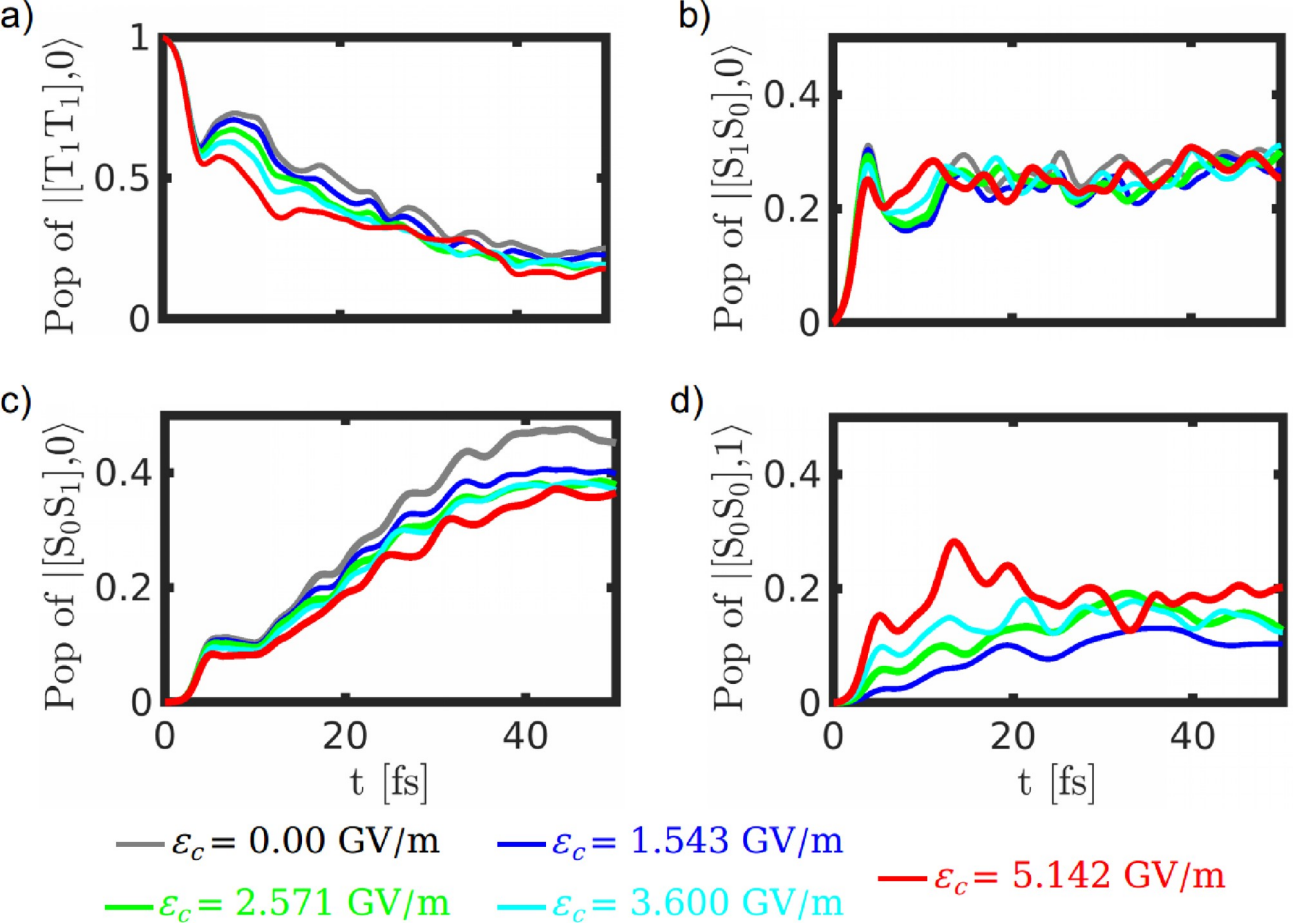
Time evolution for the population of a) $|\left[T_{1}T_{1}\right],\left.0\right\rangle$ , b) $|\left[S_{1}S_{0}\right],\left.0\right\rangle$ , c) $|\left[S_{0}S_{1}\right],\left.0\right\rangle$ and d) $|\left[S_{0}S_{0}\right],\left.1\right\rangle$ states of cavity coupled naphthalene dimer at various cavity field strengths. The combined molecule‐cavity states $|M,\left.n\right\rangle$ have been denoted as a product of molecule ($|\left.M\right\rangle$ ) and cavity Fock ($|\left.n\right\rangle$ ) states.

The PESs have been represented by a two‐dimensional grid with 256 points along *Q*_1_ and 196 points along *Q*_2_. The initial condition for the dynamics simulations has been prepared by vertically placing the lowest vibrational state corresponding to the ground electronic state, $|\left[S_{0}S_{0}\right],\nu =\left.0\right\rangle$ on the [T_1_T_1_] state (violet colored filled circle in Figure 8(b)). The time evolution of the nuclear wave packet has been performed for 50 fs with a time step of 24 as by using our in‐house quantum dynamics code, QDng. The corresponding population dynamics of [T_1_T_1_], [S_1_S_0_] and [S_0_S_1_] states have been presented with grey curves in Figure 9(a), (b) and (c), respectively. We have observed two different time scales for the decay of [T_1_T_1_] state. During the initial stage of dynamics (around 5 fs), a rapid and significant decay has been observed, followed by a relatively slow population transfer (Figure 9). The two decay time scales can be explained based on the analysis of the wave packet evolution. The nuclear wave packet first moves along the *Q*_2_ mode and quickly reaches the degenerate seam of CI_10_ within 3 fs, whereas the same seam along the *Q*_1_ mode will be approached around 10 fs (Figure S11 in Supporting Information). The non‐adiabatic couplings along the *Q*_2_ mode has been observed to be large as compared to those along the *Q*_1_ (Figure S2–S7 in Supporting Information). Consequently, [T_1_T_1_] state population transfers significantly to the lower electronic states via CI_10_ seam point along the *Q*_2_ mode. Taken together, the fast and slow decay time scales corresponding to the population dynamics of [T_1_T_1_] state can be attributed to the wave packet evolution along *Q*_2_ and *Q*_1_ modes, respectively.

**Figure 10:**
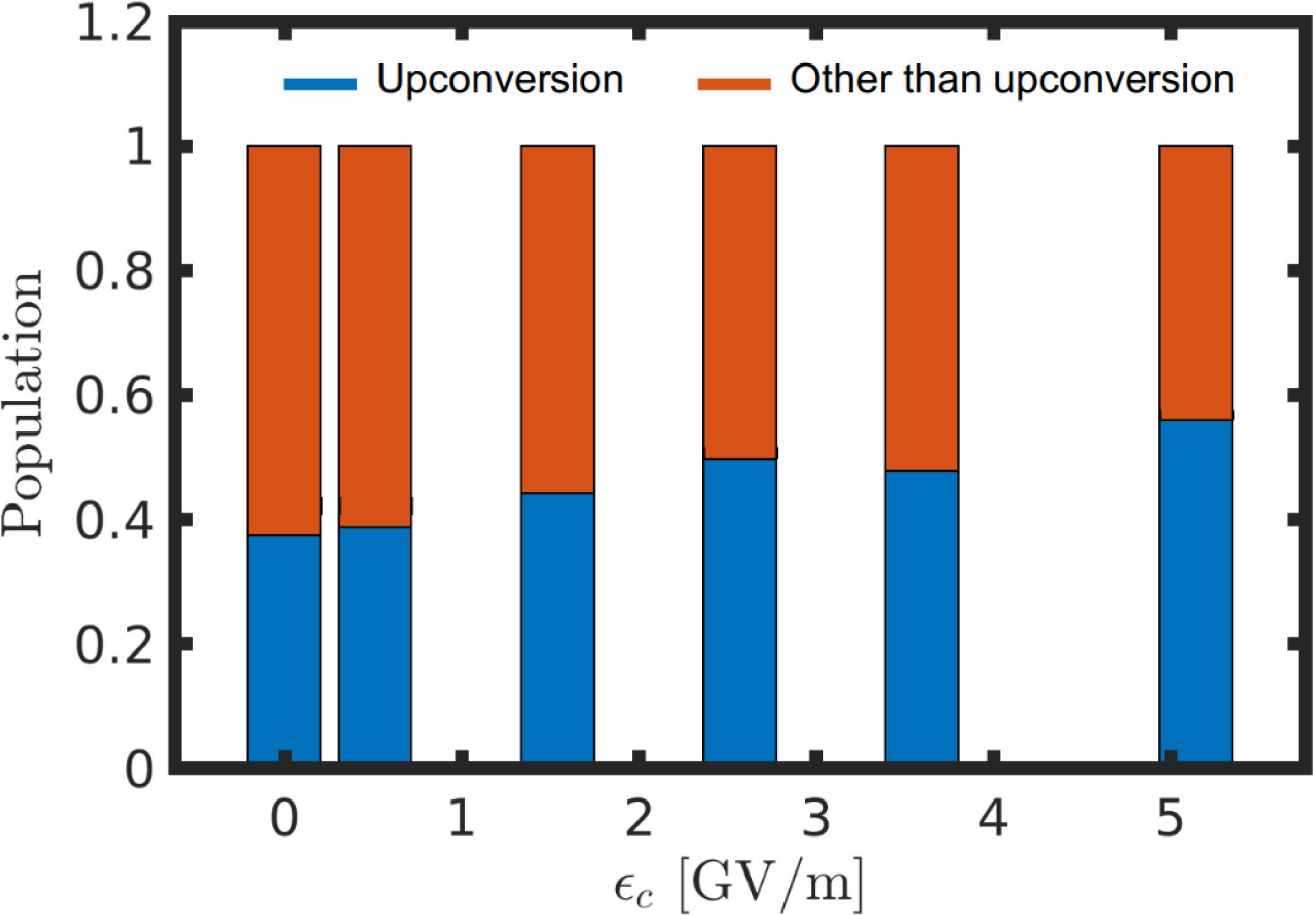
Blue area in the bar plot represents the sum of the population probabilities reaching the upconversion region on $|\left[S_{1}S_{0}\right],\left.0\right\rangle$ , $|\left[S_{0}S_{1}\right],\left.0\right\rangle$ and $|\left[S_{0}S_{0}\right],\left.1\right\rangle$ states. The red part is for the probability of the population in the remaining area of the PESs.

Now the population in the [S_1_S_0_] and [S_0_S_1_] state can access two pathways, upconversion (at the FC geometry) or the pericyclic minimum region, to proceed. Based on the branching ratio calculations by using Eq. 9, the probability of the population reaching the upconversion region has been estimated to be more than 35 % by the end of the simulation timescale (Figure 10). Here, the upconversion region is defined as the area of [S_1_S_0_] and [S_0_S_1_] PESs beyond −1 along the negative direction of *Q*_1_. Upon the wave packet reaching the reactant complex region in the singlet exciton state, the molecule ideally dissociates into two individual monomers with subsequent photon emission. However, the constructed PESs exhibit minima in the dissociative region due to the presence of the butterfly motion component in *Q*_1_. As a result, the nuclear wave packet moves back and forth between this artificial minima and the region of S_1_‐min for extended simulation timescales.

### Effects of strong coupling on the triplet fusion process

In addition to the field‐free triplet fusion dynamics of naphthalene, we also investigated how coupling to the modes of an optical cavity influences the population dynamics of TTA and the corresponding branching ratios. The cavity has been coupled to the dimer with the mode frequency of 4.63 eV, which resonates the molecule in the region of CI_10_. The cavity couplings corresponding to the different electronic states have been included in the Hamiltonian (Eq. 5) used to perform the polaritonic non‐adiabatic dynamics simulations. The coupling strength, which depends on the molecular transition dipole moment and the cavity field strength, has been varied to estimate the cavity effects on the TTA process. The electronic structure calculations on naphthalene reveal that the magnitude of transition dipole moments between ground and first singlet excited state to be relatively small (≈0.05 au). However, experimental studies commonly use acene derivatives such as TIPS‐naphthalene or TIPS‐anthracene instead of the parent molecules, and they have stronger transition dipole moments and consequent small fluorescence lifetimes.[^39^, ^74^] Ideally, the well‐functioning annihilator exhibits high fluorescence quantum yields, due to which the most of the upconversion generated excited state molecules emit photons of higher energy than the incident light, by avoiding the other competing pathways. Therefore, we have considered the larger transition dipole moments and multiplied the dipole moments with a factor of 10, to counteract the small dipole moments of naphthalene. The cavity field strength has been varied between 0.51 and 5.14 GV/m.

The population dynamics of cavity‐molecule dressed states at various cavity field strengths are shown in Figure 9(a)–(d), and the corresponding branching ratios are displayed in Figure 10. Here we have used a product basis to represent the dressed states, i. e., $|\left[T_{1}T_{1}\right],\left.0\right\rangle$ , for instance, refers to the triplet‐triplet state with zero photon whereas $|\left[S_{0}S_{0}\right],\left.1\right\rangle$ denotes the ground state with one photon. It is clear that the [T_1_T_1_] state decay rate increases significantly by increasing the cavity coupling strength. Interestingly, the rise of the population in the [S_0_S_1_] state decreases with an increase in the coupling strength, whereas the time evolution curve for [S_1_S_0_] state is unchanged. This suggests that the [T_1_T_1_] state population directly transfers to the $|\left[S_{0}S_{0}\right],\left.1\right\rangle$ state, which increases with the increase in field strength (Figure 9(d)). This is particularly beneficial, because the polaritonic state efficiently emits photons depending on the cavity lifetime. On the other hand, the [S_1_S_0_] or [S_0_S_1_] states have the possibility of dimerization along with the fluorescence. The direct population transfer to the polaritonic state via TTA process has also been observed in a recent experimental study where an endothermic TTA reaction was converted into an exothermic one by using optical cavities.^[59]^ The branching ratio calculations suggest that the population probability reaching the upconversion region of naphthalene increases to more than 50 % for the largest coupling strength.

## Conclusions

The [T_1_T_1_] state of naphthalene annihilates into the singlet excited states [S_1_S_0_] and [S_0_S_1_] through the surface crossings CI_10_ and CI_01_, respectively (Figure 5). These degenerate points act as departure positions on the PESs from which the population branches either towards the upconversion region or towards the pericyclic minimum on the first excited state. The former channel leads to the formation of FC geometries on the singlet exciton states ([S_1_S_0_] and [S_0_S_1_]) whereas the latter channel forms the excimer minimum. The fluorescence emission from the singlet exciton state produces the photon higher in energy than the incident light and this explains the photon upconversion due to the TTA process. The second channel forming the pericyclic minimum reduces the upconversion efficiency by non‐radiatively decaying to the ground state via an avoided crossing with the ground state or S_1_/S_0_ CI (Figure 5 and 6). We have developed a two‐dimensional PES model to describe the triplet fusion of naphthalene. The two reaction coordinates corresponding to the PESs of reduced dimensionality involve intermolecular stretching, butterfly motion, and symmetric ring stretching (Figure 7). The constructed PESs exhibit degenerate seams between the adjacent electronic states (Figure 8(a) and (b)). Our nuclear wave packet dynamics simulation performed on the computed PESs suggests that 35 % of the [T_1_T_1_] state population reaches the photon upconversion region while the remaining population ends up in the region of pericyclic minimum (Figure 10).

Coupling the molecule to an optical cavity increases the upconversion efficiency to more than 50 %. Furthermore, the polaritonic simulation results have shown a direct population transfer route from the [T_1_T_1_] state to the polaritonic state, which rationalizes the similar observation in an experimental study of cavity assisted TTA.^[59]^

Further extension of the current work is to incorporate the interaction between electronic states of different spin multiplicities and the cavity decay effects on the TTA generated upconversion process. The treatment of these effects in an extended dimensional PESs is beyond the scope of this paper and will be of future interest.

## Supporting Information Summary

Molecular orbitals involved in the active space, vertical excitation energies, Non‐adiabatic couplings, transition dipole moments, snapshots of nuclear wave function, and Cartesian coordinates for the optimized critical points.

## Conflict of interest

The authors declare no conflict of interest.

1

## Supporting information

As a service to our authors and readers, this journal provides supporting information supplied by the authors. Such materials are peer reviewed and may be re‐organized for online delivery, but are not copy‐edited or typeset. Technical support issues arising from supporting information (other than missing files) should be addressed to the authors.

Supporting Information

## Data Availability

The data that support the findings of this study are available from the corresponding author upon reasonable request.
