# Supplementary material for: Triplet‐triplet Annihilation Dynamics of Naphthalene
Source: Chemistry. 2022 Jun 21;28(40):e202200781. doi: 10.1002/chem.202200781 (PMC9401077; doi:10.1002/chem.202200781)
Supplement: Supplementary file 1 — Supporting Information [file CHEM-28-0-s001.pdf]

Chemistry–A European Journal

Supporting Information

Triplet-triplet Annihilation Dynamics of Naphthalene

Mahesh Gudem* and Markus Kowalewski*

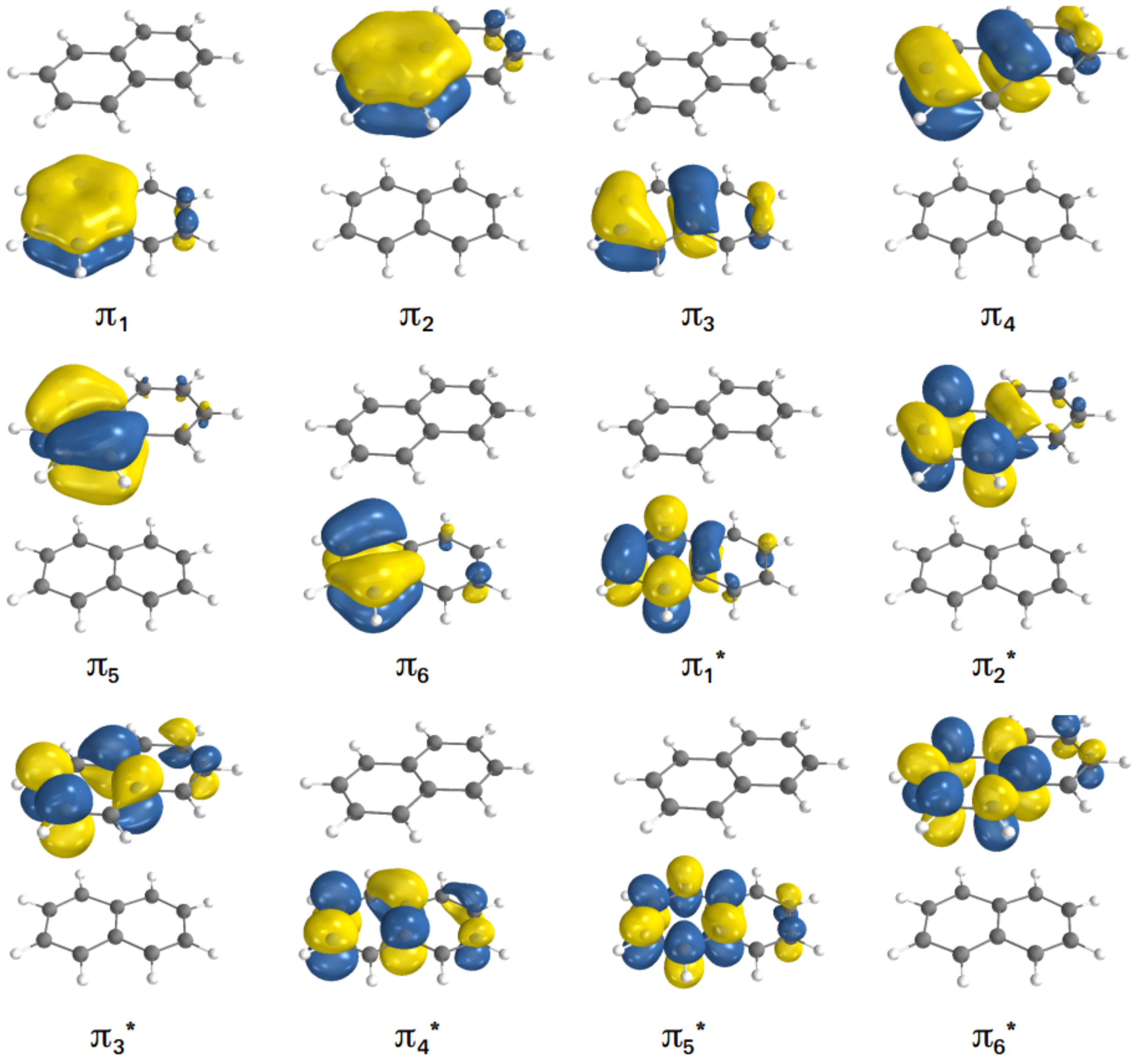

Figure S1: Molecular orbitals of naphthalene dimer considered in cas(12-in-12) active space used for the CASSCF and CASPT2 calculations.

Table S1: Vertical excitation energies (eV) of naphthalene monomer and dimer computed at CASSCF, CASPT2 and MRCI[a] methods along with 6-31G** basis set

Naphthalene monomer				Naphthalene dimer		
State	CASSCF	CASPT2	MRCI	State	CASSCF	CASPT2
S_1	5.023	5.057	5.049	$[S_1S_0]$	5.055	5.067
T_1	3.650	3.929	3.792	$[S_0S_1]$	5.076	5.088
$2xT_1$	7.300	7.858	7.584	$[T_1T_1]$	7.352	7.853

[a] MRCI method was used for monomer calculations only

Table S2: Vertical excitation energies (eV) of naphthalene monomer and dimer computed at CASSCF, CASPT2 and MRCI[a] methods along with 6-311++G** basis set

Naphthalene monomer				Naphthalene dimer		
State	CASSCF	CASPT2	MRCI	State	CASSCF	CASPT2
S_1	4.978	4.993	4.998	$[S_1S_0]$	5.017	5.004
T_1	3.624	3.896	3.763	$[S_0S_1]$	5.042	5.032
$2xT_1$	7.249	7.793	7.526	$[T_1T_1]$	7.312	7.787

[a] MRCI method was used for monomer calculations only

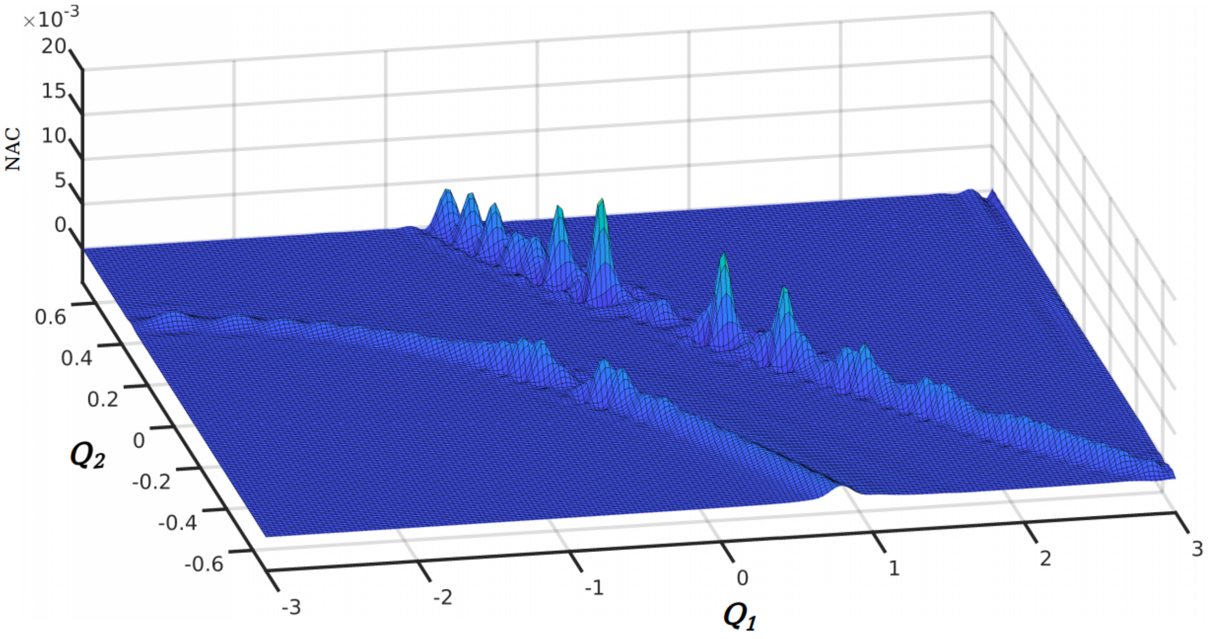

Figure S2: Mass-weighted non-adiabatic couplings between $[T_1T_1]$ and $[S_1S_0]$ states along Q_1 mode, in the span of Q_1 and Q_2 coordinates computed at CASSCF/6-31G** level of theory.

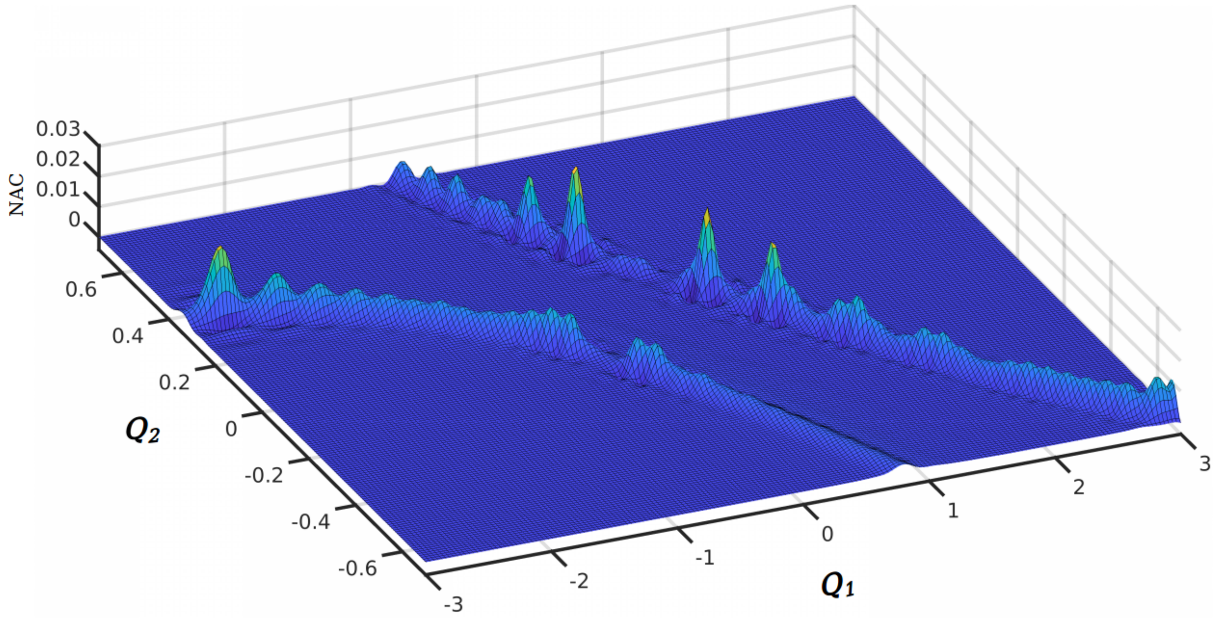

Figure S3: Mass-weighted non-adiabatic couplings between $[T_1T_1]$ and $[S_1S_0]$ states along Q_2 mode, in the span of Q_1 and Q_2 coordinates computed at CASSCF/6-31G** level of theory.

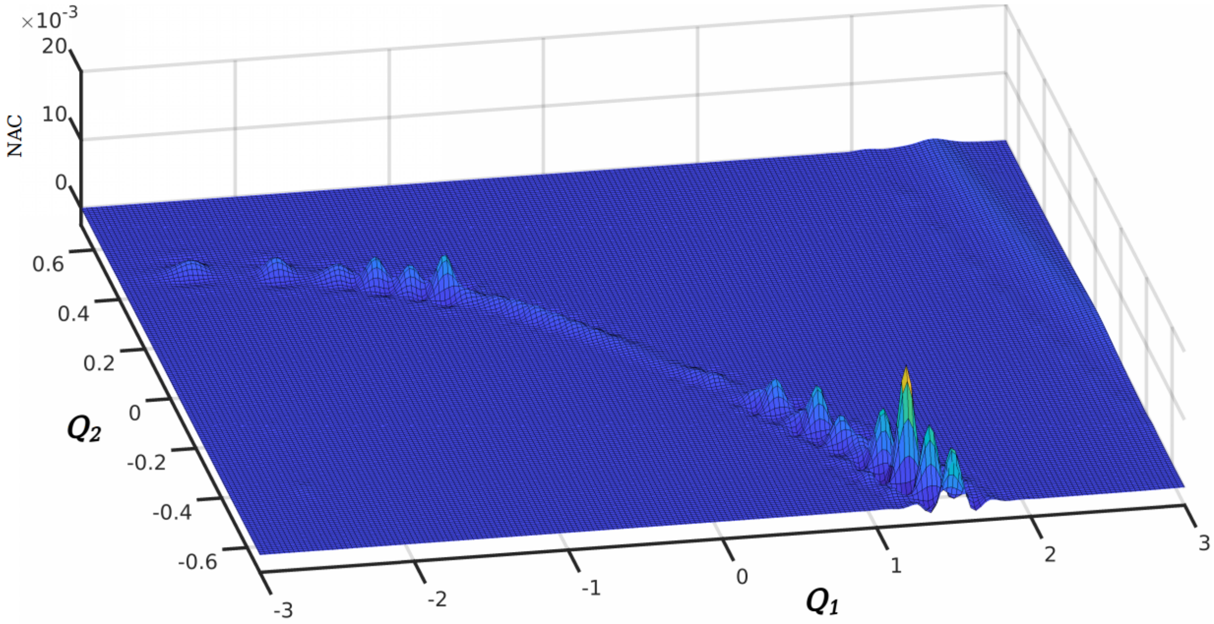

Figure S4: Mass-weighted non-adiabatic couplings between $[S_1S_0]$ and $[S_0S_1]$ states along Q_1 mode, in the span of Q_1 and Q_2 coordinates computed at CASSCF/6-31G** level of theory.

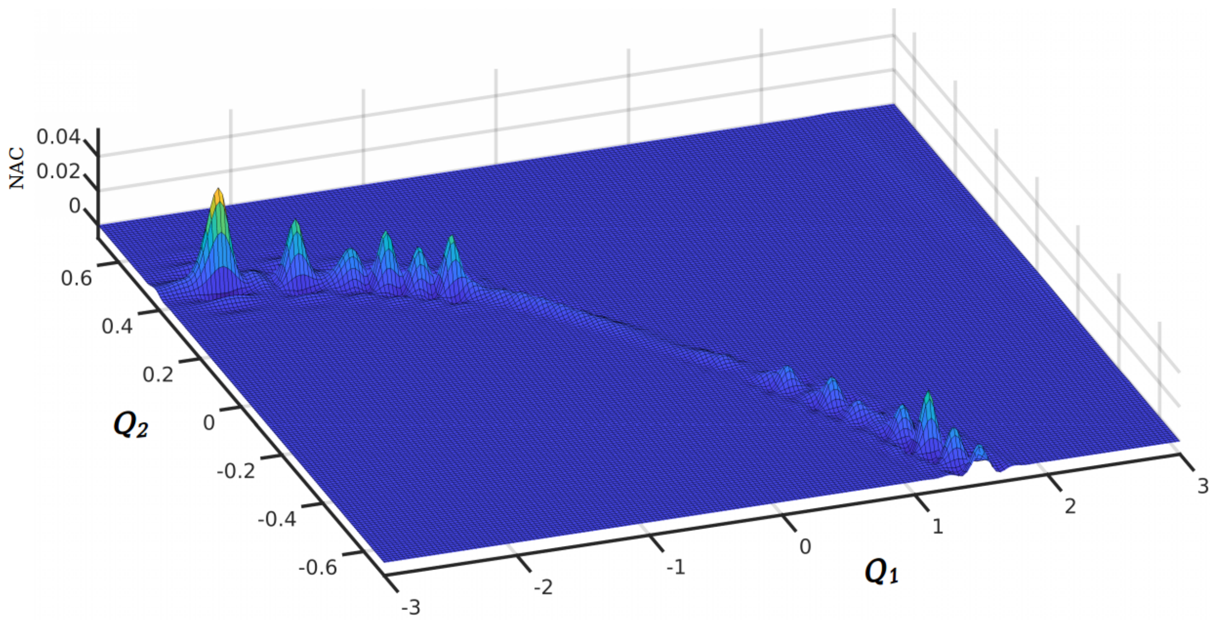

Figure S5: Mass-weighted non-adiabatic couplings between $[S_1S_0]$ and $[S_0S_1]$ states along Q_2 mode, in the span of Q_1 and Q_2 coordinates computed at CASSCF/6-31G** level of theory.

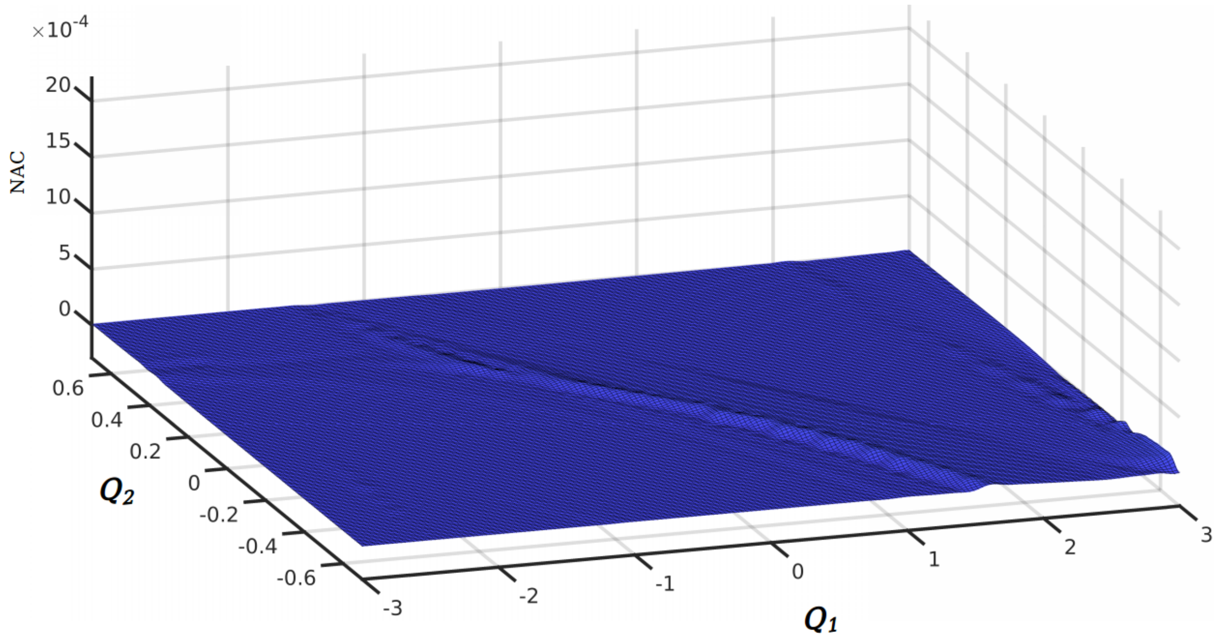

Figure S6: Mass-weighted non-adiabatic couplings between $[T_1T_1]$ and $[S_0S_1]$ states along Q_1 mode, in the span of Q_1 and Q_2 coordinates computed at CASSCF/6-31G** level of theory.

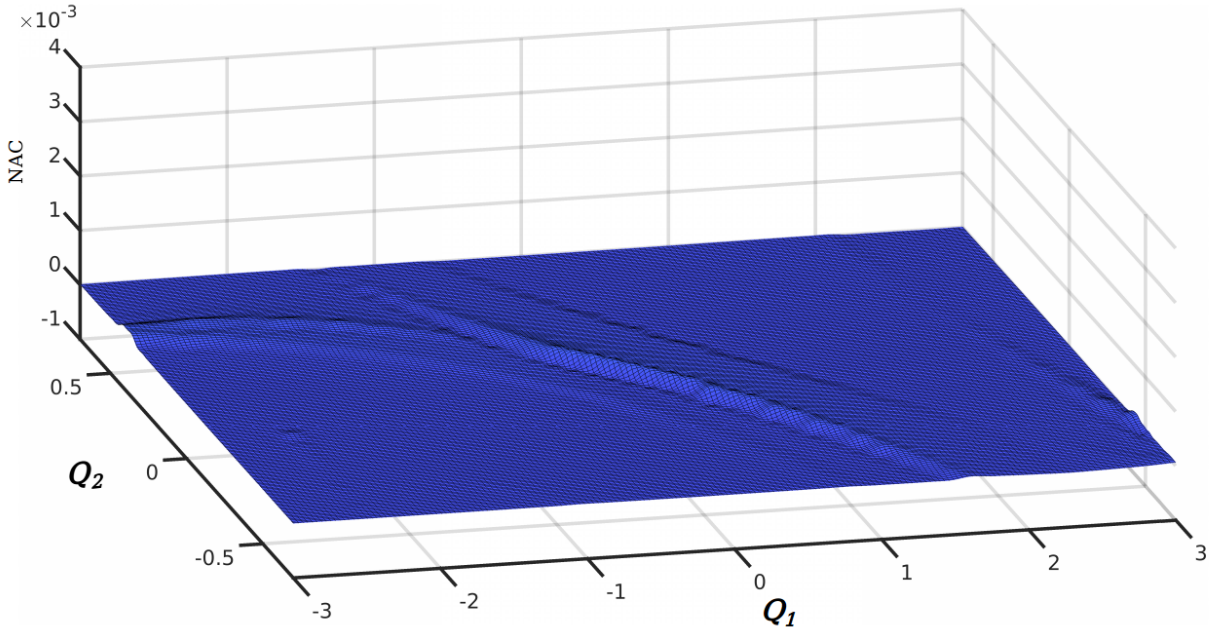

Figure S7: Mass-weighted non-adiabatic couplings between [T_1T_1] and [S_0S_1] states along Q_2 mode, in the span of Q_1 and Q_2 coordinates computed at CASSCF/6-31G** level of theory.

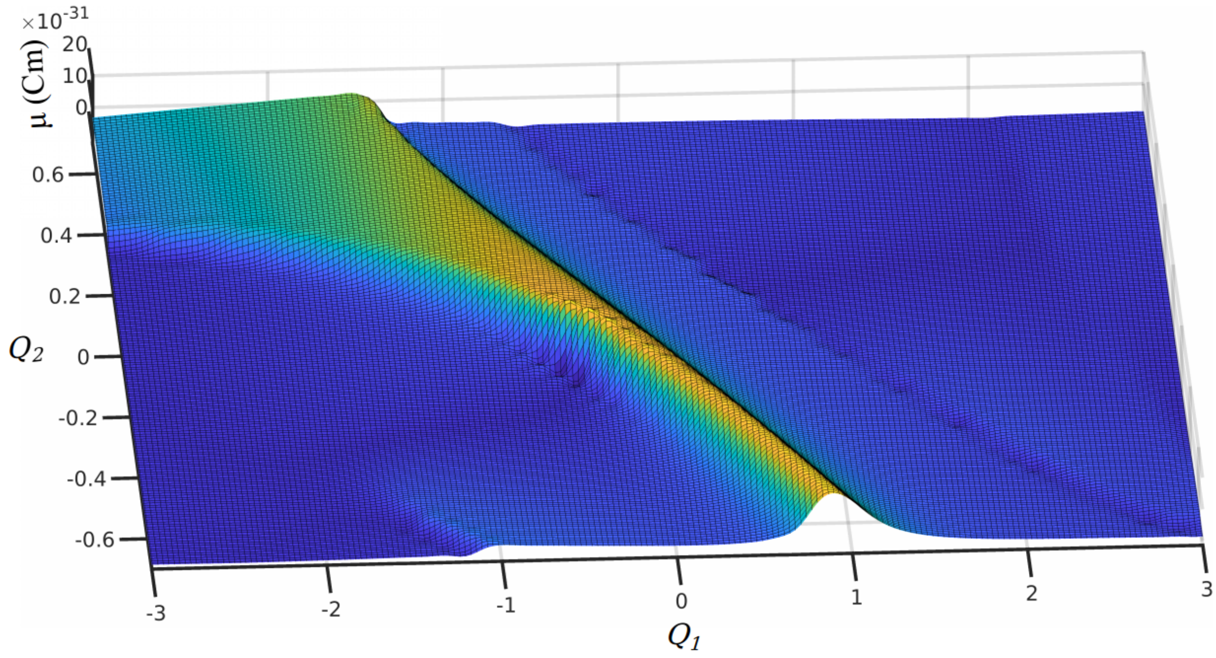

Figure S8: 2D-surface plot for the X-component of the transition dipole moment between [T_1T_1] and [S_0S_0] states computed at CASSCF/6-31G** level of theory.

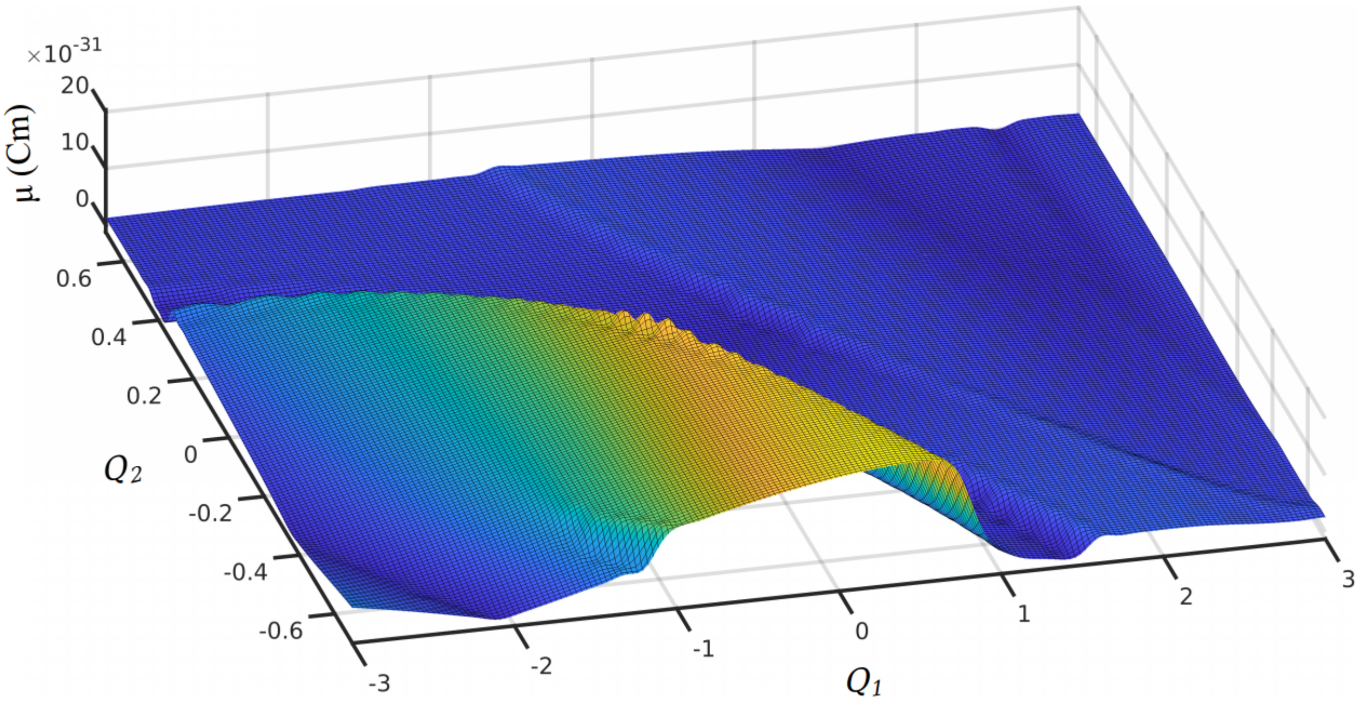

Figure S9: 2D-surface plot for the X-component of the transition dipole moment between [S_1S_0] and [S_0S_0] states computed at CASSCF/6-31G** level of theory.

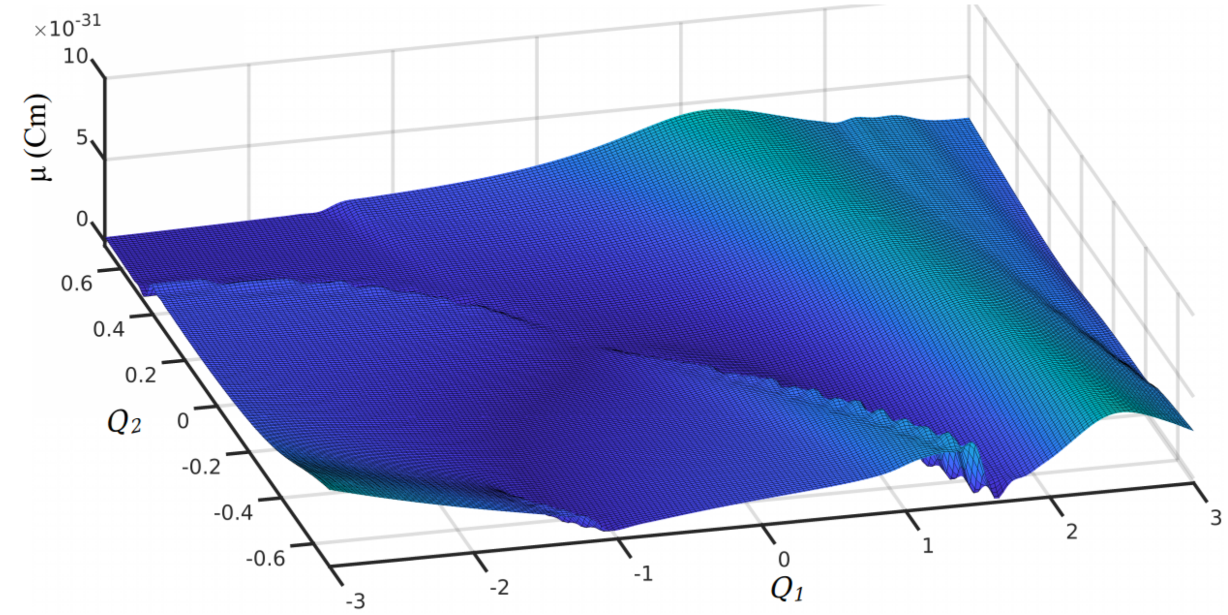

Figure S10: 2D-surface plot for the X-component of the transition dipole moment between [S_0S_1] and [S_0S_0] states computed at CASSCF/6-31G** level of theory.

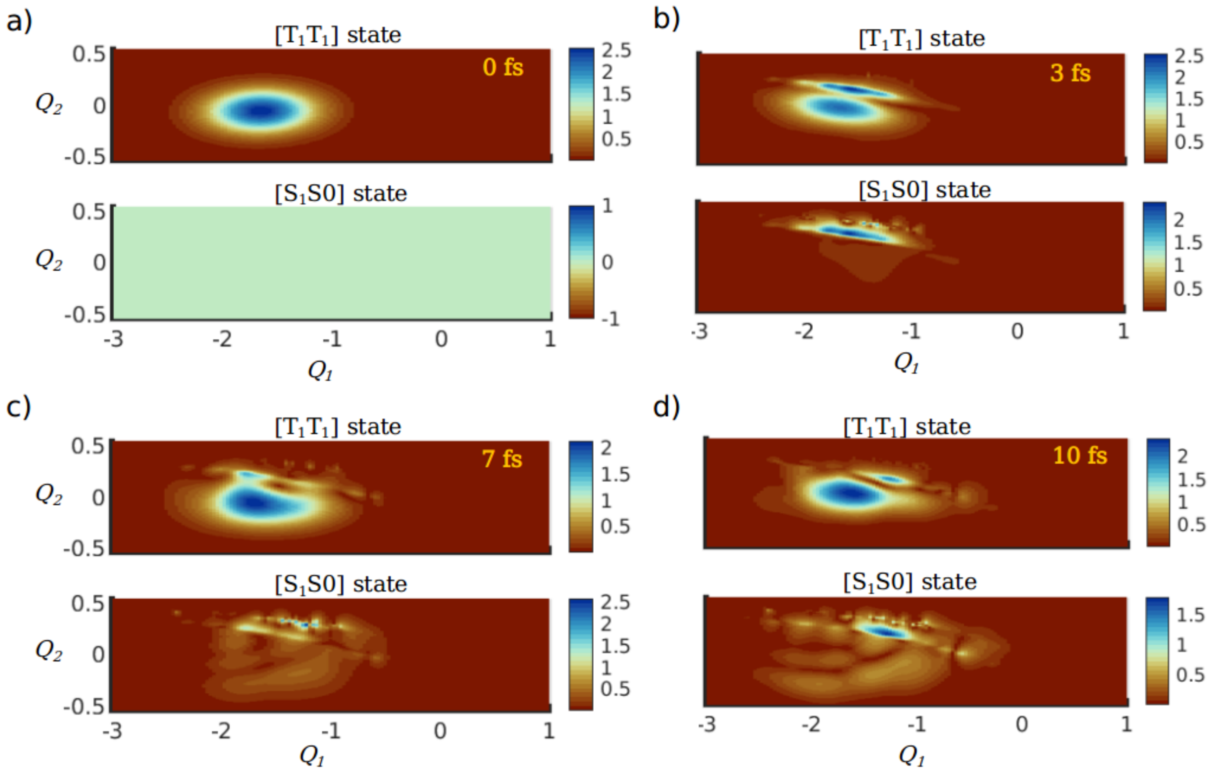

Figure S11: Time evolution of the nuclear wavepacket on $[T_1T_1]$ and $[S_1S_0]$ states for the first 10 fs.

Cartesian coordinates for optimized critical point of naphthalene dimer

S_0-crossed optimized geometry obtained at CASSCF/6-31G**			
C	0.0000000000	0.0000000000	0.0000000000
C	0.0000000000	0.0000000000	4.0072380000
C	0.0000000000	2.7402536074	4.5854856827
C	1.5360316560	2.1488687303	0.9305714871
C	2.1452661284	0.8684674856	0.7862735997
C	1.3718511748	-0.2134893864	0.3188449637
C	-0.5623604523	1.2483401989	0.1260454283
C	0.2152945339	2.3362035751	0.5979522051
C	1.1811461272	0.6009123704	4.5317681549
C	1.1815668661	1.9805318356	4.8217235401
C	2.3735325683	2.5765760040	5.3494283850
C	3.4776438123	1.8324865609	5.5702159125
C	3.4771916714	0.4359822232	5.2772723075
C	2.3730009090	-0.1562837874	4.7762790751
C	-1.1354397277	2.1363841853	4.0989111100
C	-1.1351070814	0.7490020667	3.8054637362
C	3.5256384224	0.6455848827	1.1002210403
C	4.0802160561	-0.5752996841	0.9488310683
C	3.2970889377	-1.6704937012	0.4762929314
C	1.9930082786	-1.4972441687	0.1749382625
H	0.0074538259	-1.0507225886	3.7764436338
H	0.0031019705	3.7936154864	4.8074429725
H	2.3712159067	3.6301739202	5.5712047597
H	4.3674962156	2.2866065932	5.9699807485
H	2.3736352212	-1.2075838228	4.5464652325
H	4.3677095880	-0.1401663514	5.4575972135
H	-0.5884787986	-0.8280148497	-0.3562711946
H	2.1266600830	2.9691471359	1.2988365848
H	1.4005990401	-2.3219438164	-0.1830794793
H	3.7571726179	-2.6364655689	0.3618715819
H	4.1123427486	1.4706749310	1.4649805665
H	5.1175488245	-0.7335163972	1.1863723911
H	-2.0240822961	0.2875317852	3.4139961064
H	-2.0268127534	2.7155226730	3.9332862312
H	-0.2361678580	3.3063143446	0.7049996720
H	-1.5964883955	1.4031453722	-0.1268746205

S_0-eclipsed optimized geometry obtained at CASSCF/6-31G**

C	0.0000000000	0.0000000000	0.0000000000
C	0.0000000000	0.0000000000	3.0072380000
C	0.0000000000	2.7594307937	3.4564042120
C	0.4134411889	2.7238110480	0.4767296130
C	1.5439976428	1.8650253699	0.3148211876
C	1.3346320765	0.4937197511	0.0738873521
C	-1.0607497992	0.8740950440	-0.0422588599
C	-0.8506823589	2.2539166359	0.1986988617
C	1.2131701427	0.6405661503	3.4103772994
C	1.2116944462	2.0301694446	3.6371984447
C	2.4202319546	2.6648789713	4.0695535100
C	3.5408634609	1.9431237595	4.2803701245
C	3.5442701554	0.5358677394	4.0484109805
C	2.4284632144	-0.0883873028	3.6161920174
C	-1.1880407847	2.0929669878	3.2719196220
C	-1.1871863493	0.6947388856	3.0444636363
C	2.8881901127	2.3559642846	0.3715202748
C	3.9352656580	1.5275009167	0.1763973369
C	3.7218171263	0.1387941608	-0.0710745202
C	2.4692193337	-0.3609926369	-0.1092682030
H	0.0099726786	-1.0550234505	2.7994094425
H	0.0078806306	3.8268363493	3.5974856684
H	2.4137107959	3.7275573398	4.2436005269
H	4.4410532752	2.4232996207	4.6223030275
H	2.4338179256	-1.1490856163	3.4337784165
H	4.4482641761	-0.0231764669	4.2154091728
H	-0.1586762096	-1.0542714409	-0.1504064108
H	0.5737656851	3.7642653180	0.6958864330
H	2.3039271473	-1.4085804127	-0.2958981517
H	4.5698174325	-0.5056238686	-0.2240340036
H	3.0501648805	3.4023408097	0.5644483913
H	4.9411755667	1.9076526617	0.2099096750
H	-2.1125518528	0.1927654559	2.8246684638
H	-2.1136560380	2.6390088761	3.2235288019
H	-1.6942310960	2.9201188471	0.2384712773
H	-2.0612587145	0.5052611026	-0.1850922527

S_0-adduct optimized geometry obtained at CASSCF/6-31G**

C	0.0000000000	0.0000000000	0.0000000000
C	0.0000000000	0.0000000000	1.6482092388
C	0.0000000000	2.7631181490	1.6464084161
C	-0.0013581567	2.7610889326	-0.0017565710
C	1.2298661468	2.0741843159	-0.5701230522
C	1.2306905964	0.6875629803	-0.5689218723
C	-1.2149053227	0.7098978809	-0.5701795244
C	-1.2157323147	2.0491078495	-0.5705739332
C	1.2293800411	0.6816496348	2.2235405333
C	1.2295346792	2.0820134517	2.2222764105
C	2.3069565488	2.7682881931	2.7585187043
C	3.3798716335	2.0785014892	3.2941854651
C	3.3797039419	0.6864853644	3.2954637907
C	2.3065850450	-0.0039630204	2.7610661110
C	-1.2176989711	2.0507433543	2.2111506206
C	-1.2178752480	0.7132789463	2.2115819996
C	2.3230408200	2.7683754613	-1.1049678426
C	3.3896703068	2.0811884605	-1.6261972174
C	3.3905467788	0.6814101938	-1.6249314940
C	2.3247354522	-0.0061772070	-1.1025414237
H	-0.0073137197	-1.0401411136	1.9513853342
H	-0.0068680677	3.8036403334	1.9482779514
H	2.3075497032	3.8455544193	2.7627505575
H	4.2106300835	2.6176960170	3.7144621187
H	2.3068766292	-1.0812201283	2.7673063119
H	4.2103386347	0.1478168047	3.7166770675
H	-0.0015902686	-1.0401425588	-0.3035003950
H	-0.0044297195	3.8008322809	-0.3066089605
H	2.3247113150	-1.0830982542	-1.1044115132
H	4.2277310923	0.1464146986	-2.0380488342
H	2.3216152001	3.8452907626	-1.1087603234
H	4.2261855742	2.6164793260	-2.0402910025
H	-2.0647650999	0.1417114578	2.5492053745
H	-2.0645987762	2.6227645896	2.5479545226
H	-2.0605154104	2.6216093751	-0.9117094997
H	-2.0592116742	0.1361351728	-0.9103793999

S_1-min optimized geometry obtained at CASSCF/6-31G**

C	0.0000000000	0.0000000000	0.0000000000
C	0.0000000000	0.0000000000	2.4213131609
C	0.0000000000	2.7906545189	2.5416089072
C	-0.2990501910	2.7795419437	0.1695724859
C	0.9920017493	2.2552228208	-0.3008242880
C	1.1493219825	0.8386228050	-0.3733469953
C	-1.3017083586	0.5939230573	-0.1531296257
C	-1.4615891284	1.9553669320	-0.0642873004
C	1.2673078842	0.6817970967	2.7030756537
C	1.2674533818	2.0820218336	2.7422542825
C	2.4857524058	2.7530867702	2.9615899541
C	3.6473913185	2.0535599108	3.1390162346
C	3.6431366585	0.6489118700	3.1084663752
C	2.4701011314	-0.0214961394	2.9000455818
C	-1.2022890463	2.0584143433	2.8653062112
C	-1.2146248146	0.6940644576	2.7947973964
C	2.0378490152	3.0772794934	-0.6727851196
C	3.2547815459	2.5301495787	-1.1210435535
C	3.4017598024	1.1745795098	-1.1806350486
C	2.3421879952	0.3265757337	-0.8054064419
H	0.0018112352	-1.0741246773	2.4567611144
H	0.0049518350	3.8524797393	2.7196297022
H	2.4948853109	3.8292354524	2.9908076281
H	4.5711670754	2.5796133969	3.3058791262
H	2.4606706519	-1.0980148704	2.8705279328
H	4.5605440438	0.1063030802	3.2531007227
H	0.0960588394	-1.0571133550	-0.1782391751
H	-0.4235032618	3.8468412995	0.1466331532
H	2.4728195670	-0.7402563130	-0.8687465282
H	4.3278310898	0.7437097108	-1.5192920681
H	1.9222192118	4.1464553594	-0.6173036457
H	4.0608319960	3.1821573304	-1.4074673548
H	-2.1208052729	0.1355362100	2.9430921327
H	-2.1093399037	2.5987536485	3.0734543635
H	-2.4383360544	2.4026363284	-0.0780599629
H	-2.1635007668	-0.0441651852	-0.2453050827

S_1/S_0 CI optimized geometry obtained at CASSCF/6-31G**

C	0.0000000000	0.0000000000	0.0000000000
C	0.0000000000	0.0000000000	2.1080744916
C	0.0000000000	2.8153312887	2.1318110086
C	-0.6716085543	2.4508103092	-1.3679553579
C	0.7228009432	2.0984533855	-1.1222905560
C	1.0602505654	0.8921652029	-0.4951430801
C	-1.3805467911	0.3357372629	-0.3791588754
C	-1.6453349253	1.5427459367	-1.1019658712
C	1.1640722935	0.7029236905	2.5755749462
C	1.2256999306	2.1018553155	2.4810632414
C	2.4288140518	2.7773836046	2.7477399475
C	3.5603043074	2.0889338460	3.0840673578
C	3.4959008710	0.6882012299	3.2211168461
C	2.3244603687	0.0186137768	3.0228622161
C	-1.1365684007	2.1877117908	1.7614375112
C	-1.1823909341	0.7275944735	1.6324199083
C	1.7713759094	2.9192174020	-1.6028899648
C	3.0736987909	2.5331288935	-1.4915739653
C	3.4067121352	1.2971851278	-0.9045079674
C	2.4157621235	0.4990349730	-0.4193082459
H	-0.1348695308	-1.0266577331	2.3999287815
H	0.0005022406	3.8892379673	2.2148147228
H	2.4549326167	3.8511552914	2.6634089866
H	4.4856655121	2.6070470205	3.2612167983
H	2.2877808204	-1.0514626737	3.1416845903
H	4.3760119890	0.1448996297	3.5208387179
H	0.2616913285	-1.0390533385	0.0391601453
H	-0.8880348279	3.3807039536	-1.8616840391
H	2.6596778138	-0.4384179848	0.0462654311
H	4.4356123780	0.9945855365	-0.8320277483
H	1.5228131480	3.8529494942	-2.0766083536
H	3.8554274524	3.1702793478	-1.8674619209
H	-2.1412687092	0.2774141033	1.8061165420
H	-2.0261882534	2.7481577816	1.5456073986
H	-2.6670489813	1.7546593455	-1.3700427049
H	-2.0782216790	-0.4796621933	-0.4558793480
